# In Vitro Strategies to Vascularize 3D Physiologically Relevant Models

**DOI:** 10.1002/advs.202100798

**Published:** 2021-08-05

**Authors:** Alessandra Dellaquila, Chau Le Bao, Didier Letourneur, Teresa Simon‐Yarza

**Affiliations:** ^1^ Université de Paris INSERM U1148 X Bichat Hospital Paris F‐75018 France; ^2^ Elvesys Microfluidics Innovation Center Paris 75011 France; ^3^ Biomolecular Photonics Department of Physics University of Bielefeld Bielefeld 33615 Germany; ^4^ Université Sorbonne Paris Nord Galilée Institute Villetaneuse F‐93430 France

**Keywords:** 3D cell culture, bioprinting, microfluidics, tissue engineering, vascularization

## Abstract

Vascularization of 3D models represents a major challenge of tissue engineering and a key prerequisite for their clinical and industrial application. The use of prevascularized models built from dedicated materials could solve some of the actual limitations, such as suboptimal integration of the bioconstructs within the host tissue, and would provide more in vivo‐like perfusable tissue and organ‐specific platforms. In the last decade, the fabrication of vascularized physiologically relevant 3D constructs has been attempted by numerous tissue engineering strategies, which are classified here in microfluidic technology, 3D coculture models, namely, spheroids and organoids, and biofabrication. In this review, the recent advancements in prevascularization techniques and the increasing use of natural and synthetic materials to build physiological organ‐specific models are discussed. Current drawbacks of each technology, future perspectives, and translation of vascularized tissue constructs toward clinics, pharmaceutical field, and industry are also presented. By combining complementary strategies, these models are envisioned to be successfully used for regenerative medicine and drug development in a near future.

## Graphical abstract

Vascularization strategies of 3D tissue engineered models are reviewed in this article. The integration of a vascular network in vitro is fundamental to achieve tissue constructs with physiological relevance. Nowadays, numerous approaches have been developed to vascularize tissue and organ models, mainly by using microfluidic technology, 3D cell culture, and bioprinting. Recent advances and the combination of these strategies are promoting a rapid translation toward the clinical and industrials fields.

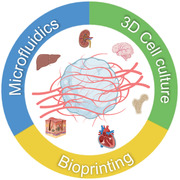

## 1 Introduction

In physiological conditions, the tissues of the human body are vascularized thanks to an abundant network of blood vessels, known as the vascular network. Human vasculature has essential biological functions, such as nutrients and gas exchange, metabolic waste removal, and homeostasis maintenance.^[^ ^1^, ^2^ ^]^ Its role is fundamental at the macro as well as at the microscale, where a diffusion limit of oxygen and nutrients has been reported to be around 200 µm,^[^ ^3^, ^4^ ^]^ meaning that the cells located farther from a capillary undergo hypoxia and apoptosis. Thus, vascularization plays a pivotal role in achieving physiologically relevant tissue and organ substitutes for tissue engineering (TE) and regenerative medicine applications. Despite the unprecedent advancements of tissue engineering in the last decades, the integration of a functional vascular network in tissue constructs still represents a challenge that hampers an efficient and fast scale‐up toward the clinical application.

In bioengineered models, the presence of vasculature would ensure the proper exchanges, preventing cellular death in constructs thicker than 200 µm and contribute in mimicking the tissue physiology and cell microenvironmental cues. Overall, a functional capillary network would allow for a long‐term maintenance of the construct in terms of viability, morphology, and functionality. Furthermore, organ‐specific vasculature has shown to strongly affect the behavior of the parenchymal cells and to drive organ‐related biological events.^[^ ^5^ ^]^ Vasculature plays a key role also in many diseases, such as cancer metastasis, atherosclerosis, or tumor angiogenesis.^[^ ^6^ ^]^ For in vitro studies, the use of vascularized models could give more realistic insights of human response to drug testing, toxicology assays, or in pathological models.^[^ ^7^ ^]^ Particularly in the pharmaceutical field, the urgent need to speed up the drug development process, lower R&D costs, and overcome the use of inadequate animal models strongly relies on the development of more predictive and clinically accurate systems.^[^ ^8^, ^9^, ^10^ ^]^ In regenerative medicine, the implantation of prevascularized constructs compared to constructs that spontaneously vascularize in situ would enhance the grafting to the host tissue and fasten its regeneration. Moreover, although the successful implantation of thin constructs like skin has been reported, the formation of abundant and functional vascular network is a key prerequisite for the generation of thick and metabolically active organs, such as liver, heart, or kidney.^[^ ^2^ ^]^ In fact, the host vasculature needs time to integrate and vascularize the implanted tissue and the use of avascular scaffolds could be inefficient due to the impossibility to be instantly perfused. The implantation of prevascularized scaffolds would thus represent one of the most favorable strategies for regenerative medicine purposes.

**Figure 1:**
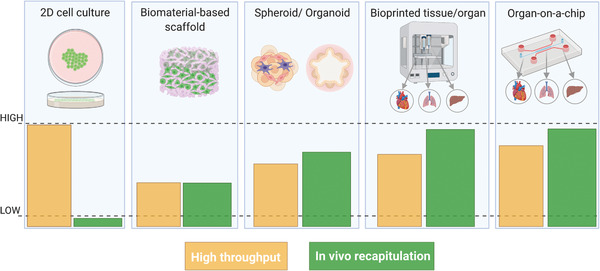
Evolution of tissue engineering platforms from 2D to 3D models. The bottom panel shows the comparison of model throughput versus physiological relevance: the in vivo recapitulation increases when moving from 2D cell cultures to 3D models and the throughput of complex models can be enhanced by means of automated bioprinting processes or parallel microfluidics. Created with BioRender.com.

Many efforts have been conducted over the past years to build 3D physiologically relevant models that could fully recapitulate the tissues and organs functioning. The traditional 2D cell culture systems on polystyrene surfaces, which have been the gold standard of in vitro models for many decades, are unable to mimic the in vivo conditions. Tissue engineering has thus developed a plethora of 3D cell culture models, which have proven to be more physiologically relevant compared to 2D cell culture, providing accurate results in biological studies, such as in vivo‐like cell viability, morphology, differentiation, and proliferation, as well as cellular response to stimuli, protein synthesis, and drug metabolism (**Figure** **1**).^[^ ^11^ ^]^

In recent decades, some research lines have thus moved from culturing of single cell types on flat and rigid substrates, to the coculture of cells, first in 2D (i.e., Transwell systems) and later in 3D, with the introduction of spheroids and organoids models. Complex physiological conditions, such as blood flow, oxygen gradients, or mechanical stimuli, can be mimicked nowadays by using microfluidic devices, that allow for perfusion of cells by means of microchannels networks. In parallel, new biomaterials have been developed to mimic the cell niche, with advancements from 2D culture on extracellular matrix (ECM) gels (i.e., Matrigel) to 3D scaffolds with tunable physical–chemical and mechanical properties.^[^ ^12^, ^13^, ^14^ ^]^ These systems have been extensively used as in vitro models consisting of multiple cell types and the combination with bioreactors has allowed researchers to provide the cells with physiological‐like biochemical and mechanical cues. Recently, these in vitro models have often adopted the emerging strategy of 3D bioprinting to engineer more complex systems, eventually replacing the conventional fabrication methods. The synergistic use of these technologies would allow for a precise control of the cell culture conditions and the microenvironment and it would represent a key strategy to engineer biostructures that mirror human tissues and organs while ensuring high throughput, fundamental for the translation of these models toward their application in industrial and clinical settings. Nevertheless, lacking or inefficient perfusion and vascularization remains one of the main limitations of tissue engineered constructs as the need for vascularization exists from the moment the tissue‐engineered constructs are assembled in vitro, to the moment when they are implanted in a patient.^[^ ^15^ ^]^

In this review, we discuss the latest advancements on vascularization strategies in tissue engineering, focusing on different approaches, namely, organs‐on‐a‐chip (OOaC), spheroids, organoids, and 3D bioprinted tissues. After a brief overview of the physiological properties of the vascular network, we describe the fabrication techniques used to engineer prevascularized 3D physiologically relevant tissue and organ models. Finally, we critically discuss the current technical limitations and evaluate some perspectives for industrial and clinical applications.

## 2 Physiological Properties of the Vascular Network

**Figure 2:**
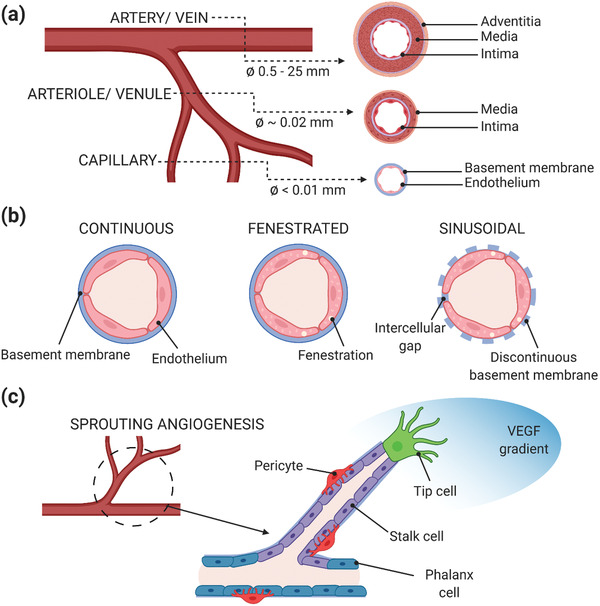
Physiological properties of the vascular network. a) Anatomical properties and dimensions of the human vasculature. b) Phenotypic heterogeneity of organ‐specific endothelium. c) Differentiated role of endothelial cells during angiogenesis. Created with BioRender.com.

The vasculature is a network of blood vessels consisting of the arterial system, the venous system, and the microcirculation (**Figure** **2**a). The arterial system, composed of arteries and arterioles, distributes oxygenated blood from the lungs while the venous system, composed of veins and venules, returns low oxygenated blood to the heart. Separating these two systems is the microcirculation, where nutrients and cellular wastes exchange is carried out by the capillaries. The distinct anatomy and size of the blood vessels are dictated by the different physiological functions they play. To withstand high blood pressures and shear stress, the larger vessels, namely, arteries and veins, are composed of three layers. The external layer, called tunica adventitia, is mainly composed of collagen and nerve fibers, with a protective and support function. The middle layer, tunica media, is composed of smooth muscle cells (SMCs) and elastic connective tissue, responsible for vasodilation and vasocontraction. The inner layer, tunica intima, is the lumen wall, lined with endothelial cells (ECs) and surrounded by a thin basement membrane.^[^ ^16^, ^17^ ^]^ The arteries and veins are large diameter vessels, ranging from 25 mm for the aorta and about 2 mm for the pulmonary veins to hundreds of micrometers for the smallest arteries and veins. While moving down into the vascular tree, the blood pressure decreases and less elasticity is needed: that is why arterioles, with a size of 10–100 µm, are composed of the tunica media and intima only and the capillaries (less than 5 µm) are composed of a single ECs monolayer. It is interesting to notice that with the decrease of the vessels size, the vascular wall also becomes thinner. At the tissue level, the anatomy is extremely complex: in healthy conditions, the capillary density is about 300–400 capillaries mm^−3^ in skeletal muscles and above 2000 capillaries mm^−3^ in myocardium, brain, liver, and kidney.^[^ ^18^ ^]^ Furthermore, the parenchymal tissues are composed of cells at high concentration, of about 10^5^ cells mm^−3^.^[^ ^19^, ^20^ ^]^ Due to its direct contact with blood, the endothelium participates in numerous physiological functions including selective barrier membrane, thrombosis prevention, blood pressure regulation, and angiogenesis.^[^ ^21^ ^]^ Although ECs in different regions of the body fulfil similar physiological demands, heterogeneity in their morphology, function, gene expression, and antigen composition has been reported.^[^ ^22^, ^23^ ^]^ Specifically, the morphology of the endothelium varies to adapt to the specific functions of their underlying tissue (Figure 2b). Most of the vessels of the brain, lungs, and skeletal muscles, present a continuous endothelium, where ECs are held together by tight junctions and a continuous basement membrane, allowing mainly for water and ion exchange. For organs that are involved in filtration and secretion (i.e., exocrine and endocrine glands, intestinal villi, kidney glomeruli, choroid in the eyes, and a subpopulation of renal tubules), the endothelium is fenestrated. These fenestrations, or pores, exist along with tight junctions in the endothelial lining, and their permeability can vary depending on the underlying tissue needs. For the vessels in the liver, spleen, and bone marrow, the endothelium is sinusoidal or discontinuous, where the lining has larger fenestration (100–200 µm), extensive intercellular gaps, and an incomplete basement membrane.^[^ ^21^ ^]^

For the development of more biomimetic vascularization strategies, we summarize here the main aspects of the two key biological processes through which neovascularization occurs: vasculogenesis and angiogenesis. Vasculogenesis is the process in which de novo blood vessels are generated from endothelial precursors, the angioblasts, in the embryo. Once the primitive vascular network is formed, more blood vessels arise from pre‐existing ones and expand through the angiogenesis process. During angiogenesis, ECs are activated through a complex cascade of proangiogenic signals and undergo division, sprouting, branching, and lumen formation to form a network of arteries and veins. Currently, most vascularization approaches intended for clinical applications focus on the latter phenomenon. ECs demonstrate a structural and functional heterogeneity during angiogenesis, when they differentiate into two phenotypes, known as tip cells and stalk cells. Tip cells produce filopodia, which explore and perceive local signals from the environment, while guiding new vessel sprouts and forming connections with neighboring cells to build vessel loops.^[^ ^24^, ^25^, ^26^ ^]^ In contrast, stalk cells follow tip cells and proliferate to support sprout elongation and lumen morphogenesis and secrete basement membrane components, which further stabilize newly formed vessels (Figure 2c).^[^ ^27^ ^]^ The phenotypic differentiation of ECs is a transient and reversible process, modulated by complex signaling pathways, as the interplay between the vascular endothelial growth factor (VEGF) and Notch signaling.^[^ ^28^, ^29^ ^]^ Tip cell migration is regulated by VEGF gradients while the Notch signaling is essential for stalk cell barrier function, polarity, and lumen formation. New vascular network connections are then stabilized through the recruitment of pericytes and vascular smooth muscle cells, followed by the deposition of ECM. Once the vessels have been perfused, ECs switch to quiescent state (phalanx phenotype), where they are immobile and nonproliferating and promote vascular stability through increased cell adhesion and reduced response to VEGF signals. Nevertheless, quiescent ECs maintain their plasticity to sense and respond to angiogenic signals.^[^ ^30^ ^]^ We refer the reader to existing reviews for a detailed overview of the angiogenetic process.^[^ ^27^, ^31^, ^32^ ^]^

## 3 Requirements for the Fabrication of Engineered Vascularized Tissues

Based on the morphological and physiological aspects illustrated so far, the engineering of functional vascularized constructs should fulfill several parameters:

i) The artificial vessels should have circular cross‐section to guarantee optimal cell seeding and physiological‐like shear stress, fundamental to maintain healthy endothelial phenotype.^[^ ^26^, ^33^, ^34^ ^]^
ii) The bioengineered vascular network should be branched and multiscale as it is in vivo, with larger vessels branching into capillaries to ensure a proper blood flow and gas and nutrients exchange at the microscale. The presence of large vessels (hundreds of micrometers) is also required when the artificial network needs to be surgically anastomosed to the host vasculature.^[^ ^3^ ^]^
iii) For vessels other than capillaries, a multilayered structure should be recreated in vitro and include not only the endothelium composing the tunica intima but also the other cellular components as the SMCs. Coaxial technology holds great promise for the fabrication of the different vessel layers, as we will illustrate in Sections 4.1.1 and 4.3.2.
iv) The tissue construct should take into account the organ‐specific morphology of the vascular endothelium (i.e., continuous, fenestrated or sinusoidal ECs), which regulates the barrier properties and the interaction between the parenchymal tissue and the blood.^[^ ^35^ ^]^ This prerequisite would necessarily require an accurate selection of cell sources, preferring primary cells over cell lines, further complicating the challenge.
iv) The in vitro vasculature microenvironment should integrate basement membrane proteins, as laminin and collage type IV, and other ECM components (fibronectin, glycosaminoglycans, …),^[^ ^14^, ^36^ ^]^ which actively influence the endothelial barrier function, differentiation, and proliferation during angiogenesis as well as tissue maintenance and remodeling.^[^ ^37^, ^38^, ^39^, ^40^, ^41^ ^]^
v) The in vitro vasculature should be perfused to ensure adequate cell survival and tissue functioning. The perfusion parameters of the vascular network should mirror the hemodynamics and blood flow properties:^[^ ^42^ ^]^ pulsatile flow should be applied for vessels mimicking the arteries and laminar flow in the microcirculatory system, with shear stresses below 10 dyne cm^−2^, values have shown to influence ECs cytoskeleton remodeling and nitric oxide levels.^[^ ^43^ ^]^ The mechanical properties of the surrounding tissue and ECM components should be designed to match the physiological values.^[^ ^44^, ^45^, ^46^, ^47^ ^]^
vi) The prevascularized model should mimic the in vivo capillary density and cellular concentration to respect the 200 µm diffusion limit and build functional dense and highly vascularized tissue substitutes or in vitro platforms.

## 4 Vascularization Approaches for Physiologically Relevant 3D Models

In this section, the fabrication strategies to prevascularize 3D physiologically relevant tissues are illustrated, classifying the vascularized models in microfluidic‐based, 3D cell culture (spheroids and organoids), and 3D bioprinted constructs. The fabrication methods described here, the features of each 3D approach, and their applications are summarized in **Table** **1**. It is worth highlighting that some of these approaches are used also as fabrication strategies for other models; in particular, bioprinting is currently used for engineering microfluidic platforms and 3D cell cultures and microfluidic devices have been used for culturing and vascularizing spheroids and organoids. Here, the vascularization strategies of each model are discussed separately while the recent trend toward the combination of these techniques is discussed in Section 4.4 about hybrid strategies.

**Table 1:** Fabrication strategies for each vascularized 3D model, comparison of their properties, and main applications. SL: soft lithography; T: templating; B: bioprinting; EB: extrusion‐based; DB: droplet‐based; LAB: laser‐assisted; Vat‐P: vat photopolymerization. Created with Biorender.com

	Vascularized 3D model			
	Microfluidic‐based	3D cell culture		Bioprinted
Fabrication strategy	Soft lithography Templating Bioprinting	ECM scaffolding Hanging drop Low adherent plate Bioreactor‐based	Magnetic levitation Micropatterning Microfluidics Bioprinting	Extrusion‐based Laser‐based Inkjet‐based Vat photopolymerization
Vessel geometry	Rectangular cross‐section (SL) Straight circular channels (T) Branched microvasculature (T, B)	Capillary‐like structures in vitro		Tubular interconnected channels
Vessel dimensions	Hundreds to tens of micrometers (>30 µm)	Hundreds to tens of micrometers		Tens of micrometers (Vat‐P) Hundreds of micrometers (EB, DB) Hundreds to tens of micrometers (LAB)
Microenvironment	Possibility to integrate ECM proteins and growth factors	Cell–cell interactions Possible to integrate ECM proteins and growth factors		Possibility to integrate ECM proteins and growth factors and to print multiple cell types
Advantages	Physiological shear stress In vivo‐like cues (oxygen gradient, mechanical stimuli, …) Modular and multiorgan platforms Integration of sensors for monitoring on‐chip	Scalable to various cell culture platforms Patient specific Vascular network mimicking in vivo complexity and architecture		Fast method Multiple cell/materials (EB) Thick constructs (EB) Low cost (E, DB, Vat‐P)
Disadvantages	Use of nonbiomimetic polymers (SL) Expensive equipment for fabrication Multistep production Need for external pumps	Limited diffusion and nutrient transport as size increases Difficult to manipulate Need for a large number of cells to generate substantial quantity of tissue		Low resolution (EB) Limited cell density Cell sedimentation during the bioprinting process (EB) Bioink printability limits mechanical properties (EB) High cost (LAB)
Application	Disease modeling Drug discovery and development Personalized medicine	Disease modeling Drug discovery and development Personalized medicine		Clinical application Disease modeling Drug discovery and development Personalized medicine

### 4.1 Vascularization Techniques for Microfluidic‐Based Models

In the last decade, microfluidics has emerged as relevant technology to build 3D in vitro microphysiological systems for the study of human pathophysiology and drug development.^[^ ^48^, ^49^ ^]^ The capability of engineering perfusable channels in microfluidic devices makes this technology particularly interesting to generate vascular networks in vitro and important efforts have been conducted to recreate and integrate microvasculature in OOaC models.^[^ ^50^ ^]^ The recent combination with tissue engineering approaches and biomaterials has accelerated the transition from traditional nonbiomimetic materials (glass, silicon, and polydimethylsiloxane (PDMS)) and 2D cell culture to 3D ECM‐like hydrogel‐based platforms.^[^ ^17^, ^51^ ^]^ Microfluidic‐based vascular models have been used to study the response of endothelium to a plethora of stimuli under both physiological and pathological conditions,^[^ ^6^, ^52^, ^53^ ^]^ the interaction between endothelium and parenchyma in organ‐specific vascular platforms and to understand key factors in vasculogenesis and angiogenesis processes.^[^ ^43^, ^54^ ^]^ Microfluidics has been used as well for investigating the interaction between blood cells (platelets, leukocytes, and red blood cells) and vasculature and their response to mechanical or biochemical cues, which cannot be studied with static traditional in vitro platforms.^[^ ^55^, ^56^, ^57^, ^58^ ^]^

#### 4.1.1 Strategies to Create Vasculature On‐Chip

**Figure 3:**
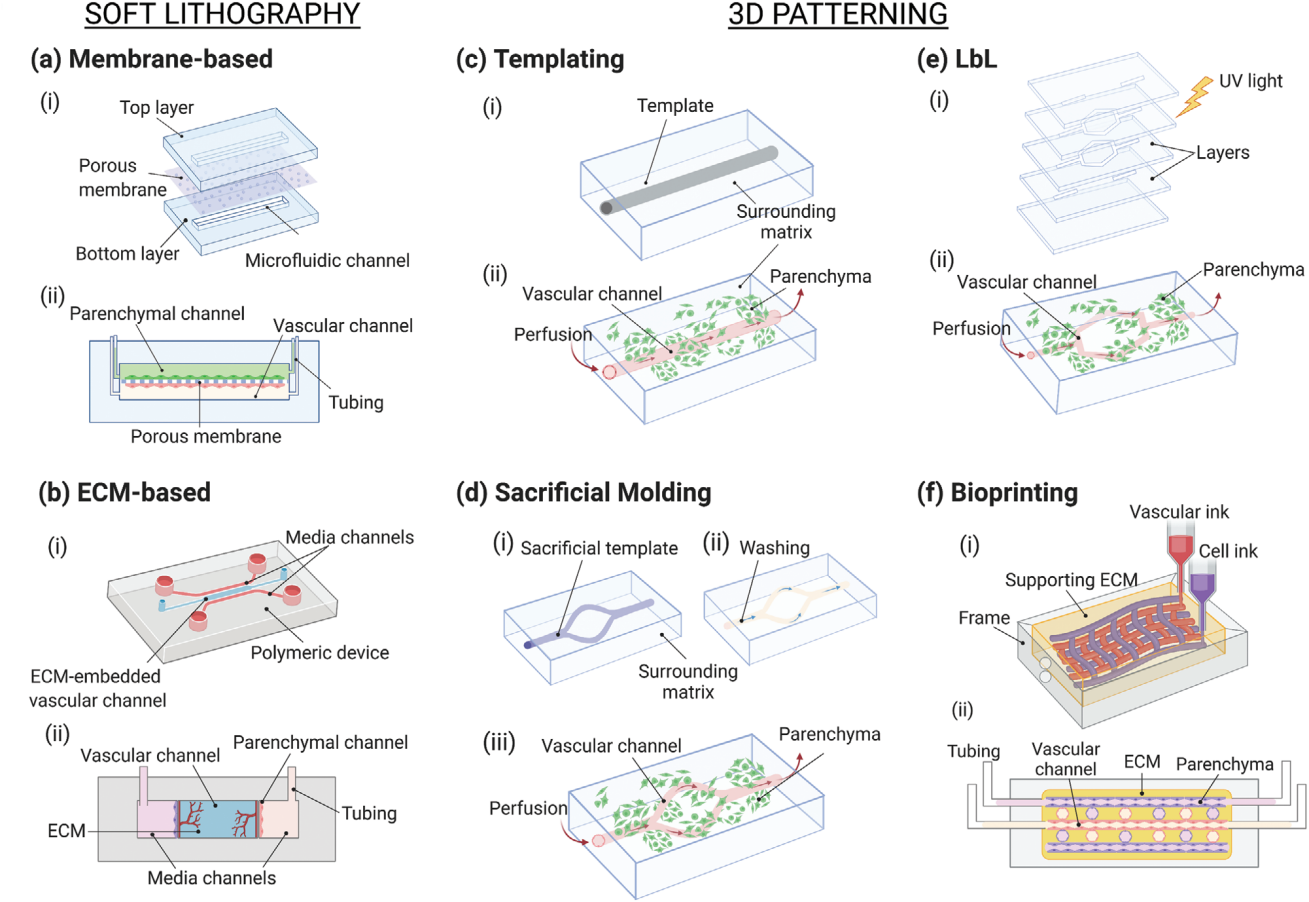
Schematic of the strategies used to vascularize microfluidic‐based models. a,b) Soft lithography and c–f) patterning. a) Membrane‐based vascularized device: i) the fabrication process consists of assembling the microfluidic layers and a porous membrane and the assembled chip with the typical sandwiched structure. b) ECM‐based microfluidic platform: i) the chip usually contains one or more channels filled with ECM proteins that ii) embed the parenchymal and vascular components. c) Templating: i) a matrix is casted around the template equipment (needle, fiber), which is ii) subsequently removed to form the channel. d) Sacrificial molding: i) the patterned template is fabricated and encased in the surrounding matrix, ii) the template is removed, and iii) the device is seeded and perfused. e) Layer‐by‐layer: the modular layers are assembled, for instance, i) by photocrosslinking before ii) the device seeding. f) Bioprinting for microfluidics: usually performed on ECM matrix—eventually bioprinted—in which vascular and parenchymal inks can be used to i) build the tissue before ii) perfusion of the device. Created with BioRender.com.

The vascularization approaches on‐chip are commonly classified based on the fabrication method into two main categories, namely, prevascularized patterning methods and self‐vascularization approaches.^[^ ^1^, ^59^ ^]^ Prevascularized patterning methods consist of engineering polymeric or biological materials to create a vascular‐like network on‐chip, which can provide physical support and guidance for cells. To form the vascular component, cells are seeded or patterned and cultured in these preformed channels (**Figure** **3**). In the self‐vascularization approach, ECs are embedded in a matrix and supplied with biological, chemical or mechanical cues to induce spontaneous morphogenesis of the vascular network. Self‐vascularized microfluidic platforms are commonly used to study vasculogenesis and angiogenesis processes in vitro (see Section 2) and they become particularly significant in the context of vasculature‐related diseases, such as cancer metastasis or atherosclerosis.^[^ ^6^, ^48^ ^]^ Comprehensive reviews on the topic are available.^[^ ^1^, ^17^, ^48^, ^60^, ^61^ ^]^ In this section, we provide an overview of the main prevascularization patterning strategies used for fabricating vascularized microfluidic platforms, focusing on relevant organ‐on‐a‐chip models integrating vasculature and discussing the current bottlenecks of this approach.

##### Soft Lithography Techniques

The mimicry of the vascular interface in vitro has been mainly achieved by using microfluidic platforms produced by soft lithography. This approach involves the production of a silicon or glass mold containing the microchannel features by photolithography and using it as stamp to pattern PDMS devices by replica molding. The device is then sealed by bonding it to a substrate to create perfusable channels (Figure 3a).^[^ ^5^ ^]^ Despite the lack of a proper 3D lumen and geometrical similarity to in vivo vasculature, these models have demonstrated to be efficient platforms to build a functional organ‐vasculature interface, showing significant advantages compared to static 2D models.

###### Vascular Interface on a Membrane

The visionary work of Ingber's group led to the development of the most used organ‐on‐a‐chip model nowadays. They reproduced the air–liquid interface (ALI) of the lung by culturing alveolar epithelial cells and human pulmonary microvascular ECs on two sides of a porous 10 µm thick PDMS membrane in a two‐channel PDMS device.^[^ ^62^ ^]^ Cyclic mechanical strain was applied to mimic physiological breathing by lateral vacuum channels. This simple yet functional platform was used to recreate a long‐term model (>2 weeks) of the ALI, showing in vivo‐like barrier permeability, enhanced production of surfactants by the epithelium when exposed to air and endothelium alignment under mechanical stretching. Exposure to cytokines and nanoparticles showed the active role of vasculature and mechanical forces under inflammatory conditions, underlying the need to integrate these components to build complex in vitro platforms capable of recreating physiological organ functions.^[^ ^53^ ^]^

**Figure 4:**
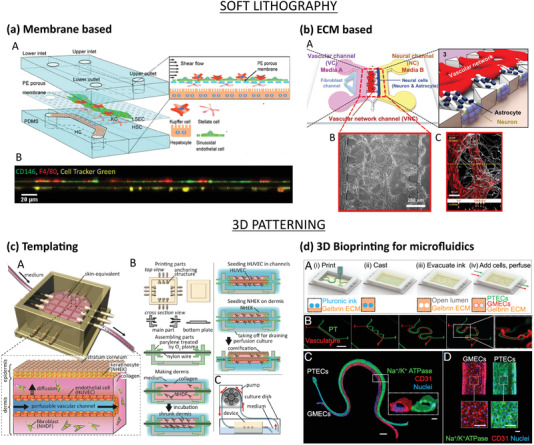
Microfluidic‐based vascularization strategies: soft lithography (top) and 3D patterning (bottom). a) Liver sinusoid on‐chip fabricated by soft lithography. LSECs and KCs were seeded on the apical side of a PE membrane while HSCs on its basolateral side and HCs on the PDMS substrate (top). Lateral view of the sinusoidal endothelium (bottom): LSECs (green) and KCs (red) on the top and HSCs (yellow) on the bottom of the membrane. Reproduced with permission.^[^ ^73^ ^]^ Copyright 2017, The Royal Society of Chemistry. b) ECM‐based vascularized BBB platform: A) HUVECs and fibroblasts were seeded in the vascular channel (VC), and neural cells (astrocytes and neurons) were seeded in the neural channel (NC). The formation of vascular network in the central vascular network channel (VNC) ensured a direct interface between the capillaries and the astrocytes through astrocytic endfeet (B,C: ECs stained in red, astrocytes stained in white). Adapted with permission.^[^ ^66^ ^]^ Copyright 2017, Springer Nature. c) Skin‐equivalent platform generated by templating: A,B) The culture device was 3D printed and filled with collagen and fibroblasts to form the dermis layer. After removal of the nylon wires, the hollow channel was seeded with HUVECs to form the capillary, and keratinocytes were cultured on the top of the dermis and exposed to liquid–air interface for cornification of the epidermal layer. C) Perfusion of the device via peristaltic pump. Reproduced with permission.^[^ ^74^ ^]^ Copyright 2017, Elsevier Inc. d) Hybrid strategy: 3D printed vascularized proximal tubule model. A,B) The colocalized vascular and renal channels are both 3D printed by using a Pluronic F127‐based fugitive ink within an ECM solution and different designs can be easily printed. C,D) The construct is then seeded with epithelial (green) and endothelial (red) cells. Reproduced with permission.^[^ ^75^ ^]^ Copyright 2019, PNAS.

This pioneering platform paved the way for the study of tissue‐vasculature interactions in organ‐specific models such as kidney,^[^ ^63^, ^64^ ^]^ brain and blood–brain barrier (BBB),^[^ ^65^, ^66^, ^67^ ^]^ heart,^[^ ^68^, ^69^ ^]^ gut,^[^ ^70^, ^71^ ^]^ and liver.^[^ ^72^, ^73^ ^]^ Recently, a liver sinusoid on‐chip was built by integrating four primary hepatic cell types from the same murine source.^[^ ^73^ ^]^ Liver sinusoidal endothelial cells (LSECs) and Kupffer cells (KCs) were cultured on the apical side of a porous polyester membrane to mimic the sinusoidal interface. Hepatic stellate cells (HSCs) were cultured on the basolateral side and hepatocytes (HCs) were seeded on the PDMS bottom channel to recreate the Disse space and the parenchymal tissue respectively (**Figure** **4**a). Shear stress was applied in the device and imaging analyses confirmed the formation of a discontinuous endothelium composed of fenestrated LSECs, typical of in vivo liver sinusoid.^[^ ^5^ ^]^ Results showed that the presence of nonparenchymal cells (NPCs) and shear stress enhanced hepatocytes functionality and metabolism compared to HCs static monoculture and neutrophil recruitment resulted to be higher when LSECs were cultured with the other NPCs under flow. Despite the use of murine cell source and the short‐term evaluation, this model reveals the synergistic effect of mechanical cues and paracrine pathways in regulating liver metabolism and its response to inflammatory conditions.

###### Multiorgan‐on‐a‐Chip (MOC): A New Promising Tool for Drug Development

The growing need for accurate and reliable in vitro models for drug screening and development has led to the design of MOC platforms (also known as body‐on‐a‐chip), that allow for the study of pharmacokinetic–pharmacodynamic (PK–PD) pathways of drugs and interactions among organ equivalents.^[^ ^76^ ^]^ The integration of vasculature is fundamental due to the active role of microvascular circulation in maintaining homeostasis.^[^ ^48^, ^59^ ^]^ Novak et al. have recently engineered a vascularized eight‐organ‐on‐a‐chip (BBB, brain, skin, lung, heart, liver, intestine, and kidney) coupled with liquid‐handling robotics and in situ microscopy that enabled automated culture, perfusion and control on‐chip.^[^ ^77^ ^]^ Interestingly, the device used a universal blood‐like medium for the vascular compartment and a specific medium for each organ. Although the vascular component was part of each organ platform, it was not included in the connections between chips. Schimek et al. lined uniformly the connecting tubes of a MOC with primary human dermal microvascular endothelial cells (hDMECs) under pulsatile shear stress and created branching microvessels of 40 µm in diameter by two‐photon laser ablation technique.^[^ ^78^ ^]^ Similarly, PDMS tubes with tunable diameter and thickness that can mimic different blood vessel types have been endothelialized and coupled to MOC platforms and the exposure to drugs showed the formation of a responsive endothelium.^[^ ^79^ ^]^

###### ECM‐Based Microfluidic Devices

Standard lithographic processes lead to rectangular or squared cross‐sectioned channels, a geometry that has been proven inadequate to build functional microvasculature in vitro and to model phenomena such as coagulation.^[^ ^80^, ^81^ ^]^ Thus, channels with circular cross‐sections have been fabricated by different strategies as micromilling of metal molds,^[^ ^82^ ^]^ flow of nitrogen gas in a PDMS solution,^[^ ^33^ ^]^ reflow of positive photoresists,^[^ ^83^ ^]^ or by viscous fingering of ECM substrates, as collagen or Matrigel.^[^ ^84^, ^85^ ^]^ Moreover, in standard microfluidic devices, cells are cultured on flat substrates such as polymeric membranes or PDMS sheets. To address these limitations, microfabrication strategies have been adapted to create hydrogel‐based microfluidic platform, for instance, by molding ECM gels upon PDMS stamps,^[^ ^86^, ^87^ ^]^ or embedding hydrogels in PDMS devices (Figure 3b).^[^ ^66^, ^88^ ^]^ In a recent work, Bang et al. engineered a BBB device with contact of astrocytes and vascular network through astrocytic endfeet to overcome the lack of direct interface of the two components in common BBB‐on‐chip platforms, that hampers the achievement of in vivo‐like barrier permeability values (Figure 4b).^[^ ^66^ ^]^ The PDMS device was composed of two parallel microchannels, representing the vascular and neural compartments respectively, embedded in a fibrin hydrogel and supplied with specific medium through lateral channels. In a first step, a mixture of endothelial cells (human umbilical vein endothelial cells, HUVECs) and fibroblasts was seeded in the vascular channel and vasculogenesis was induced to create the vascular network. After 3 days, the neural channel was seeded with astrocytes and neurons and the formation of functional BBB was observed within one week. Results confirmed the growth of a functional lumen, the migration of astrocytes to form a direct contact with HUVECs, permeability values comparable to in vivo coefficients and formation of synapses.

##### 3D Patterning Methods

The recent adoption of tissue engineering fabrication methods has paved the way for engineering more sophisticated 3D in vitro vascular networks on‐chip, overcoming the main drawbacks of conventional OOaC platforms, namely, the use of nonbiomimetic materials and lack of a 3D geometrical complexity.^[^ ^89^ ^]^ Hydrogel‐based devices reproducing the role of ECM in vivo offer several advantages such as tunable mechanical properties, biodegradability, control over the cellular microenvironment, and a wide choice of materials.^[^ ^90^ ^]^ We classify below the patterning methods used for microfluidics as templating, layer‐by‐layer (LbL) manufacturing and 3D bioprinting.

###### Templating Strategies

Templating, also known as micromolding, is a subtractive technique in which a material with the desired vasculature shape is embedded in a bulk matrix and subsequently removed or dissolved to create a hollow perfusable microvasculature. Microneedles and fibers have been widely used to fabricate simple vascular geometries in gels (Figure 3c). Mori et al. used needle‐based micromolding to create a skin‐equivalent model composed of epidermal and dermal layer and perfusable vascular channels.^[^ ^74^ ^]^ A culture device was 3D printed and nylon wires (500 µm thickness) were used as channel templates. Collagen solution loaded with normal human dermal fibroblasts (hNDFs) was gelled to fabricate the dermal layer and, after removal of the wires, the vascular channel was formed by seeding HUVECs. The subsequent addition of normal human epidermal keratinocytes (NHEKs) on the top of the dermal layer and exposure to the ALI enabled the formation of the stratum corneum of the epidermis (Figure 4c). Water repellency and capacitance tests confirmed the barrier function of the epidermal layer and permeability studies on the vascular channel showed the formation of a selective barrier for the diffusion of nutrients. Percutaneous absorption studies conducted by flowing caffeine and drugs in the vasculature confirmed the adequacy of the model as a platform for vascular absorption studies, fundamental in drug and cosmetics testing.

The needle‐based vascularized platforms are mainly limited to straight channel geometries and some manufacturing steps (needle removal, stability of the gel after crosslinking) have to be taken into account during the design process. Sacrificial molding uses templating materials that are dissolved after the hydrogel bulk gelation and represents a versatile technique to create stable and more complex 3D vascular networks.^[^ ^89^ ^]^ Gelatin (Gel),^[^ ^91^ ^]^ agarose,^[^ ^92^ ^]^ alginate,^[^ ^93^, ^94^ ^]^ Pluronic,^[^ ^95^ ^]^ and poly(vinyl alcohol) (PVA)^[^ ^96^ ^]^ have been used as sacrificial materials for creating meshes either by micromolding or 3D printing (Figure 3d and Section 4.3). Vollert et al. fabricated large (15 × 25 × 3 mm^3^) perfusable engineered heart tissues for cardiac regeneration by using either straight or branched alginate fibers as lumen template.^[^ ^93^ ^]^ The tissue was composed of a neonatal rat heart cells mix (cardiomyocytes (CMs), ECs, fibroblasts, and SMCs),^[^ ^97^ ^]^ embedded in a fibrin matrix and ECs showed formation of an intima‐like layer by spontaneously covering the vessels after alginate dissolution. The engineered tissues showed contractile forces and the continuous perfusion enhanced oxygen concentration, with a significant increase in the CMs density.

To overcome the use of potential cytotoxic dissolving agents during sacrificial molding, researchers have engineered vascular templates that can be dissolved in cell media, such as Pluronic and 3D‐printed self‐standing carbohydrate glass lattices and caramel templates, which have been used to create complex hierarchical networks of tubular channels with interconnected lumens and permeable walls.^[^ ^98^, ^99^ ^]^

###### Layer‐by‐Layer Manufacturing

LbL represents a versatile bottom‐up method for manufacturing complex 3D vasculature in vitro and consists of assembling 2D prepatterned gel slabs into multilayered (modular) 3D devices (Figure 3e).^[^ ^17^ ^]^ Zhang et al. fabricated vascularized cardiac and hepatic constructs by stacking 25 µm thick poly(octamethylene maleate (anhydride) citrate) (POMaC) layers patterned by UV photolithography.^[^ ^69^, ^100^ ^]^ The presence of microholes and nanopores in the scaffold walls ensured physiological‐like mass transport and cell migration and the formation of vessels with a thickness of 2–3 cells. The use of a photocrosslinkable hydrogel provided for tunable stiffness, thus creating an anisotropic construct that closely mimics the myocardium mechanical properties. The pump‐free perfusion in vitro was performed by connecting the device to a custom‐made bioreactor and the open configuration enabled direct access to the cellular compartments by pipetting. Culturing of the vascular network with HUVECs led to formation of a functional lumen, capable to respond to angiogenic and inflammatory stimuli and compatible with human whole blood flow. By integrating liver or heart parenchymal cells embedded in ECM, functional tissue constructs were built, exhibiting metabolic response to drug administration and contractile behavior, respectively. In vivo implantation by anastomosis confirmed the nonthrombogenic properties of the device and successful angiogenesis in a rat model.

###### 3D Bioprinting for Microfluidics

Cells and hydrogels can be used as bioinks for direct fabrication on‐chip of perfusable or vascularized models with complex geometries by means of 3D bioprinting approaches (Figure 3f). Here, we consider 3D bioprinting for fabrication of microfluidic devices as hybrid strategy, discussed in Section 4.4.

Although soft lithography, templating and additive manufacturing are commonly used for fabrication of perfusable vasculature, other methods have been explored. Heintz et al. used a laser‐based degradation technique to create complex and tortuous 3D microfluidic poly(ethylene glycol diacrylate) (PEGDA) hydrogel networks from a stack image of mouse cerebral cortex vasculature.^[^ ^101^ ^]^ The high spatial resolution offered by the technique led to microvessels with a diameter of less than 10 µm and a dense network, fundamental for providing the parenchymal tissue with nutrients and oxygen within the diffusion limit.^[^ ^3^ ^]^ Interestingly, vascular microfluidic chips have been engineered by reversibly assembling explanted mouse arteries on automated platforms, showing the capability to study intact vessels functionality by performing immunofluorescence studies and quantitative analyses on‐chip.^[^ ^5^, ^102^ ^]^

**Table** **2** summarizes significant case studies for the microfluidic‐based vascularization strategies, cited or discussed in the text. Data such as channel shape, perfusion parameters and duration of in vitro studies have been reported to provide the reader with a detailed overview of different specifications and address some drawbacks, which will be discussed in the next paragraph.

**Table 2:** Summary of case studies for microfluidic‐based vascularization strategies

Vascularization method		Organ/tissue model	Vessel caliber (*d*)/channel size (*h* × *w*)	Channel shape	Chip composition	Cellular composition	Perfusion parameters	Duration of in vitro study	In vivo evaluation/drug testing	Refs.
Soft lithography		Liver	100 µm × 1 mm	Rectangular, straight	PDMS; PE (polyester) membrane	LSECs, HCs, KCs, HSCs (all primary from mouse)	0.1 or 0.5 dynes cm^−2^, syringe pump	1 day	No/No	^[^ ^73^ ^]^
		BBB	Vascular channel: 800 µm, vascular network: <50 µm 35–100 µm	Rectangular, straight	PDMS; fibrin hydrogel PDMS; fibrin‐hyaluronic acid	HUVECs, lung fibroblasts, rat cortex neural cells Brain microvascular ECs; HUVECs; pericytes; astrocytes	–	7 days	No/No	^[^ ^66^, ^88^ ^]^
		Kidney	0.2 × 1 mm (vascular), 1 × 1 mm (urinary)	Rectangular, straight	PDMS (chip, membrane)	Glomerular ECs; hIPS‐derived podocytes	0.017 dynes cm^−2^ (vascular), 0.0007 dynes cm−^2^ (urinary), peristaltic pump	8 days	No/Yes	^[^ ^64^ ^]^
		Intestine	0.15 × 1 mm	Rectangular, straight	PDMS (chip, membrane)	Capillary ECs or lymphatic microvascular ECs, Caco‐2 intestinal epithelial cells, immune cells	0.02 dynes cm^−2^	>7 days	No/Yes	^[^ ^70^ ^]^
		Multiorgan	(100 × 500 µm) to 40 µm	Rectangular, curved	PDMS	Dermal microvascular ECs	10–40 dynes cm^−2^, on‐chip micropump	Up to 4 weeks	No/No	^[^ ^78^ ^]^
Templating		Liver	300 µm (main channel) 100–1000 µm	Circular, straight Circular, curved, branched	Collagen I GelMA	HUVECs; hepatocytes (HepG2); MSCs HUVECs, hepatocytes (HepG2)	10^−1^ dynes cm^−2^, peristaltic pump 50 µL h^−1^, syringe pump	8 days 7 days	No/No No/Yes	^[^ ^19^, ^103^ ^]^
		Skin	520 µm 100–250 µm	Circular Rectangular, curved, branched	Collagen I Collagen I	HUVECs, dermal fibroblasts, epidermal keratinocytes HUVECs, iPSC‐derived ECs, dermal fibroblasts, keratinocytes	2–3 mL h^−1^, peristaltic pump Syringe pump	10 days 21 days for cornification, 2 days at ALI	No/Yes Yes/No	^[^ ^74^, ^94^ ^]^
		Heart	100–500 µm	Circular	Fibrin matrix	Neonatal rat heart cells mix	20 µL h^−1^, syringe pump	21 days	No/No	^[^ ^93^ ^]^
3D bioprinting		Urothelial/vascular tissue^a)^	≈700 µm (inner), ≈1 mm (outer)	Circular, multilayer, flexible	Bioink: PEGOA + GelMA	Urothelial: human urothelial cells + human bladder SMCs; vascular: HUVECs + human SMCs	–	14–21 days	No/No	^[^ ^104^ ^]^
		Kidney	200 µm	Circular, colocalized	Gelatin + fibrin ECM; bioink: pluronic F127 + PEO	Glomerular microvascular ECs; proximal tubule epithelial cells	0.3 dynes cm^−2^	18 days	No/Yes	^[^ ^75^ ^]^
		Heart	500 µm (200–900 µm)	Circular, hierarchical, multibranched	Agarose hydrogel, PCL network	Rat cardomiocytes	Syringe pump	5 days	Yes/No	^[^ ^99^ ^]^
		Vasculature^a)^	1.5 × 3 mm	Rectangular, straight	PMMA case, GelMA	Aortic ECs; aortic SMCs; fibroblasts	100 µL h^−1^, peristaltic pump	7 days	No/No	^[^ ^105^ ^]^
Alternative strategies	LbL	Liver/heart	100 × 50 to 100 µm	Rectangular, straight	POMaC, fibrin gel/Matrigel for parenchyma	Liver: HUVECs + hESC‐derived hepatocytes + hMSCs; heart: HUVECs + hESC‐derived CMs + hMSCs	0.6 dynes cm^−2^, bioreactor	7 days	Yes/Yes	^[^ ^69^, ^100^ ^]^
	Laser‐based	–	<10 µm	Tortuous, dense	PEGDA	Mouse brain ECs	10 µL min^−1^	11 days	No/No	^[^ ^101^ ^]^
	Explanted vessels	Brain	120 µm	Circular, physiological	PDMS	ECs, SMCs	0.5 µL min^−1^, no external pump	–	No/Yes	^[^ ^102^ ^]^

^a)^ Bioinks containing cells.

#### 4.1.2 Limitations of Microfluidic‐Based Vascularized Models

Microfluidic technology has shown great potential for the development of in vitro vascularized models for the study of the microenvironment under healthy and pathological conditions and for drugs screening and development. Soft lithography and membrane‐based models represent a landmark for recreating the vascular interface and have been used to mimic complex organ‐specific pathophysiological mechanisms. However, they fail in recapitulating a 3D microenvironment and the membranes, made usually of artificial polymers, prevent the direct interaction of the vascular and parenchymal components. The use of ECM‐based membranes or channels has allowed researchers to move toward more physiologically relevant models,^[^ ^66^, ^106^ ^]^ but still soft lithography requires expensive equipment and makes the platforms often difficult to be used by a wide end‐users range. Templating represents a straightforward method to create hollow channels in a matrix. Although the use of 3D additive manufacturing to print the sacrificial patterns has increased the potential of the technique in fabricating more in vivo‐like networks,^[^ ^94^ ^]^ the platforms are usually limited to relatively simple geometries and large vessels of hundreds of micrometers. These methods usually require several fabrication and seeding steps and the template removal step should be designed carefully to avoid device or cellular damage. Layer‐by‐layer manufacturing, offers the possibility to design more versatile and flexible platforms via a multilayer assembling process and represents a valuable technique for engineering large‐scale thick constructs.^[^ ^107^, ^108^ ^]^ However, the precise alignment of the layers often represents a critical step in the process design. Recently, 3D bioprinting has been widely used for vascularization of biomaterials and fabrication of perfusable vessels due to its scalability, versatility, wide materials selection, and precision in engineering complex 3D cell laden constructs,^[^ ^109^, ^110^ ^]^ and its combined use with microfluidics will be further discussed in Section 4.4.

### 4.2 3D Cell Culture Models: Spheroids and Organoids

Spheroids and organoids are 3D, multicellularized structures usually devoid of any exogenous materials. In the last decade, these structures have gained significant popularity in 3D cell culture research due to their ability to mimic the physiological conditions of cells in vivo. Although the two terms have been used interchangeably, there are fundamental differences and application varieties between them. Spheroids are established from simple clusters of cells, ranging from immortalized cell lines, primary cells, or fragments of human tissue.^[^ ^13^, ^111^ ^]^ Spheroid technology was developed based on the ability of cells to self‐organize during embryonic development. This self‐assembly process takes place in vitro when cells cannot attach to their biomaterial surface, hence aggregate into spherical 3D structures, namely, spheroids. Organoids are complex clusters of cells derived from stem cells such as adult stem cells, embryonic stem cells (ESCs), and induced pluripotent stem cells (iPSCs). When given a scaffolding ECM environment (usually collagen or Matrigel matrix), they self‐assemble into microscopic analogs of their parent organs.^[^ ^112^, ^113^ ^]^ As a result, organoids are widely regarded as miniature versions of organs. Organoids retain the parental organs’ genetic features over several passages, which allows for long‐term in vitro expansion of cells and guaranties long‐term viability.

Spheroids have shown potential in mimicking tumor tissues, which could help researchers develop more physiologically relevant cancer models, hence develop better cancer treatments. Vascularized spheroids, which can be achieved via coculture with ECs, have been employed as a model to study angiogenesis in vitro and as a prevascularization approach for tissue engineering applications.^[^ ^114^ ^]^ However, as spheroids are formed via cell–cell adhesion, they only transiently mimic physiological cell organization.^[^ ^111^ ^]^ In contrast, organoids formation relies on internal developmental processes, which gives rise to a higher order of self‐assembly, hence, the unique ability to recapitulate in vivo physiological functions.^[^ ^112^ ^]^ Since organoids can be derived from patient tissues, they are interesting for disease modeling, development of personalized medicine, as well as drug testing and toxicity studies (see Section 6).^[^ ^115^ ^]^

#### 4.2.1 Spheroids and Organoids Generation

Spheroids are formed by culturing cells in hanging drops, round‐bottom nonadherent or low adhesive substrates, and in suspension to induce self‐aggregation. Alternatively, spinner flask cultures can be employed to induce spontaneous cell aggregation for the fabrication of both spheroids and organoids. In this method, cell suspension is housed inside a spinner flask bioreactor with continuous mixing via a stirring bar, which generates a convectional force that induces cell aggregates formation.

Organoid fabrication methods involve formation of 3D aggregates from stem cells, followed by embedding in a biogel such as Matrigel and culturing in a specialized mixture of media and factors to obtain specific organoid generation. To date, a wide range of organoid systems including heart, lung, brain, lung, liver, kidney, intestine, retina, etc., have been developed.^[^ ^116^, ^117^, ^118^, ^119^, ^120^ ^]^

**Table** **3** summarizes the different methods for the fabrication of spheroids and organoids, their advantages, and challenges. To further explore these topics, we refer the reader to published reviews.^[^ ^121^, ^122^, ^123^, ^124^ ^]^

**Table 3:** Overview of spheroid and organoid formation methods

Method	3D culture system	Description	Advantages	Challenges	Refs.
ECM scaffolding	Organoids	Stem cells are placed in Matrigel (or ECM mix) and maintained in culture	Replicates microenvironment Observation of cell adhesion and migration	Lack of reproducibility using natural ECM Synthetic ECM requires upregulating reagents	^[^ ^117^, ^125^, ^126^ ^]^
Hanging drop	Spheroids	Cells are suspended in media droplet. Cell aggregation occurs at the air–liquid interface	Consistent Does not require ECM Possible to integrate array production	Difficulties with media change Small size Low throughput	^[^ ^127^, ^128^ ^]^
Low‐adherent surfaces	Spheroids	Cells are suspended and cultured on a low‐adherent plate, or hydrophilic substrates (i.e., hydrogel) to form aggregates	Does not require ECM Cost‐effective	Not adaptable to all cell types Heterogeneous population	^[^ ^129^ ^]^
Spinning bioreactor	Spheroids and organoids	Cell suspension is housed inside a spinner flask or a bioreactor with continuous mixing. Cell aggregation is induced by convectional force	Can generate a wide range of model sizes	Large and heterogenous structures Shear forces on cells	^[^ ^130^, ^131^ ^]^
Magnetic levitation	Spheroids and organoids	Nanoparticles are ingested by cells, which are then placed in a low‐adherent substrate. A magnet lid is used to induce cell aggregation	Does not require ECM or media	NPs are expensive and toxic	^[^ ^132^ ^]^
Bioprinting	Organoids	Additive manufacture of cytokines, cells, and ECM	Generates complex and organized structures Use of multiple cell types	Bioink selection with desired characteristics	^[^ ^133^, ^134^, ^135^ ^]^
Micropatterning	Spheroids	Microcontact printing and soft‐lithography patterning of ECM	Structure control Array production	Expensive equipment Poor reproducibility Lack of patterning efficiency	^[^ ^136^ ^]^
Microfluidics	Spheroids and organoids	3D structures housed inside microstructures, integrated with microsensors	Replicates microenvironment Allows for nutrient delivery Avoids necrosis Array production	Low cell recovery Postcell analysis challenges	^[^ ^137^ ^]^

#### 4.2.2 Strategies to Vascularize Spheroids and Organoids

Researchers have shown that the incorporation of ECs increases cell viability and functions in multicellular spheroids and enables the formation of rudimentary vascular networks within the spheroid structures.^[^ ^138^, ^139^, ^140^, ^141^, ^142^ ^]^ The concept of using spheroids containing ECs dated back in 1998 when Korff and co‐workers used EC‐covered spheroids to analyze angiogenesis in vitro: ECs on the spheroids surface exhibited quiescent phenotype, which increased their sensitivity to angiogenic stimulation and differentiation.^[^ ^142^ ^]^ The incorporation of ECs in the coculture system mimics the physiological interactions between ECs and other cell types, which consequently preserves cell viability and promotes proliferation and vascularization. Along with ECs, mesenchymal stem cells (MSCs) play a key role in the angiogenic process by facilitating blood vessel stabilization and maturation.^[^ ^143^, ^144^ ^]^ Specifically, MSCs actively participate in angiogenesis via secretion of proangiogenic factors (i.e., VEGF, MCP‐1, IL‐6, etc.) and MSC‐released paracrine factors are responsible for activation of the ECs angiogenic functions.^[^ ^143^, ^145^ ^]^ Given their multipotency, MSCs also induce direct differentiation and cell–cell interactions with endothelial lineage, suggesting that MSCs could be used to facilitate vascularization in spheroids and organoids.^[^ ^144^ ^]^ For example, spheroids fabricated using only MSCs was found to generate vascularized spheroids with improved osteogenic differentiation and bone formation.^[^ ^146^ ^]^ Similarly, when human mesenchymal stem cells (hMSCs) were cocultured with HUVECs, the resulting spheroids formed capillary‐like vessels, hence improved adipogenic differentiation upon transplantation.^[^ ^147^ ^]^

**Figure 5:**
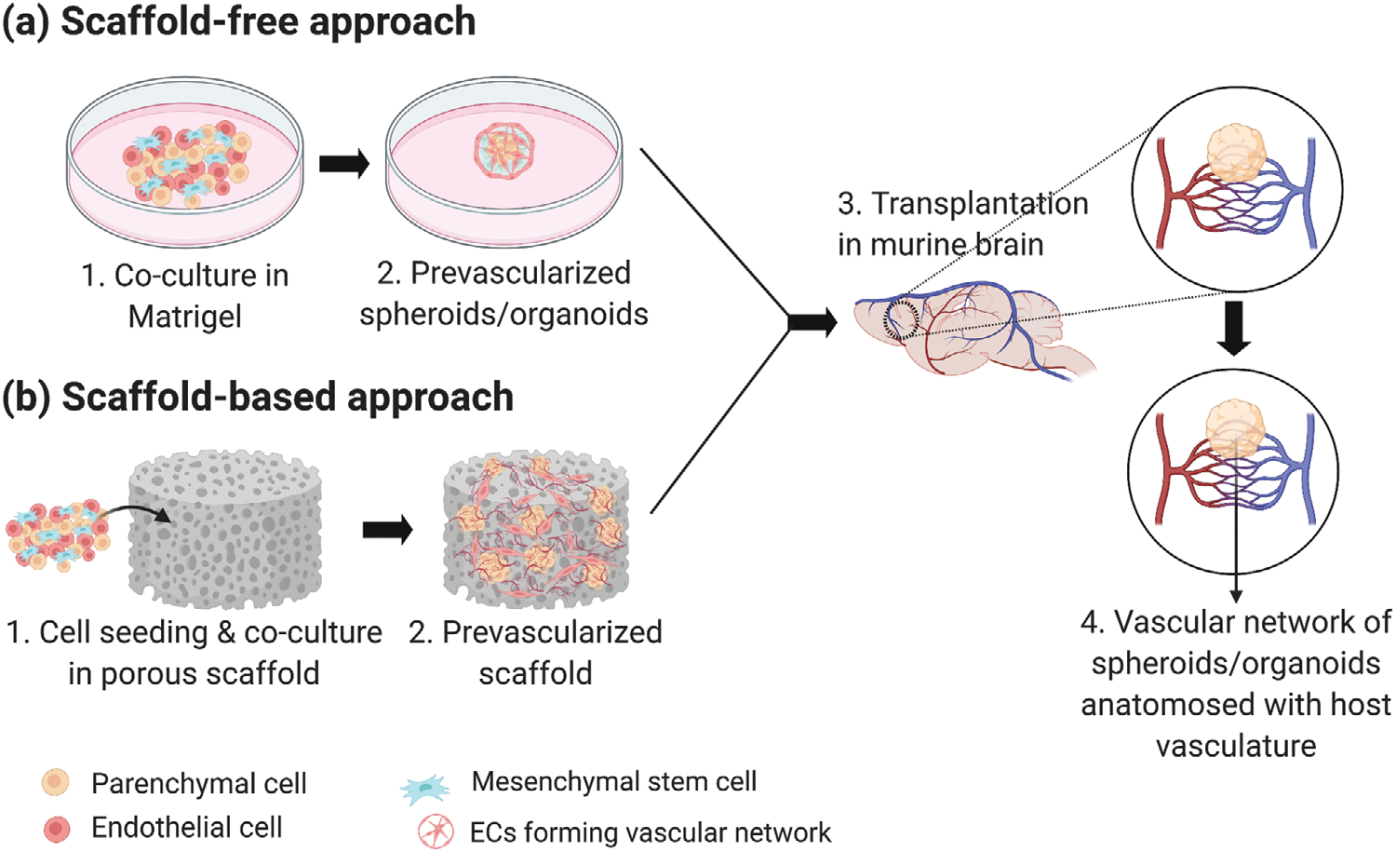
Schematic of the strategies used to vascularize 3D cell culture models. a) Scaffold‐free approach: Coculture with ECs/MSCs to form prevascularized network. b) Scaffold‐based approach: Coculture with ECs/MSCs in porous biomaterials. Both (a) and (b) can be followed by spontaneous vascularization via in vivo transplantation in highly vascularized organ such as the brain. Created with BioRender.com.

In general, the strategies used to vascularize spheroids and organoids are conducted in two steps: first, the spheroids/organoids are formed by coculturing parenchymal cells with ECs and/or MSCs to induce prevascularization in vitro. Then, spontaneous vascularization is induced via in vivo transplantation in highly vascularized regions such as skin, liver, heart, lung, or brain (**Figure** **5**). The coculture step can be achieved either via i) scaffold‐free approach (Figure 5a), or ii) scaffold‐based approach, with incorporation of a biomaterial as instructive guide (Figure 5b), discussed in the next paragraphs. Here, we consider low‐adherent substrates, hanging‐drop technique (in the case of spheroids) and Matrigel (in the case of organoids) as scaffold‐free since they do not require additional procedures, as compared to biomaterial‐based scaffolds, which are synthesized in the lab. Alternative options to standard culture techniques are the incorporation of 3D printing, bioprinting, and microfluidic platforms to form vascularized spheroids and organoids. We refer the integration of several techniques as hybrid strategies for vascularization of in vitro models, including 3D cell cultures, which are discussed in Section 4.4.

##### Vascularization of Spheroids: Scaffold‐Free Approach

Multicellular spheroids consisting of hDMECs, human osteoblasts (HOBs), and normal hNDFs were reported to have promising potential as vascularization units for bone tissue engineering.^[^ ^139^ ^]^ Spheroids have been generated using the low‐adherent surface fabrication method. Coculture spheroids with round morphology formed after 72 h, with endothelial cells showing CD31 markers. Additionally, the presence of microvessels formation within the coculture spheroids suggests prevascularization/intrinsic vascularization. The prevascularized spheroids were then harvested and transplanted into the dorsal skin of immunodeficient mice for 2 weeks. Intravital analysis of the transplanted spheroids revealed the presence of vessel‐like structures: human microvascular networks grew outside of the spheroids border and eventually connected to the host vasculature.

Cocultures of ECs with other organ‐specific cell types such as dental pulp stem cells (DPSCs), rat neonatal cardiomyocytes (RNCMs), rat hepatocytes, and human brain astrocytes and pericytes have also shown vascularization potential.^[^ ^128^, ^129^, ^140^, ^148^ ^]^ In Dissanayaka's study, DPSCs were cocultured with HUVECs and results showed microvascular networks forming within the in vitro spheroids.^[^ ^129^ ^]^ Upon in vivo transplantation, the lumens of the grafts were lined with ECs and graft vessels and mouse vessels were both present in the implanted site, suggesting integration of prevascularized spheroids into the host vasculature. This study finding highlights the potential of EC‐incorporated spheroids as functional vascularized units that can promote successful dental pulp regeneration.

Bhang and colleagues were among the first researchers to demonstrate the feasibility of generating spheroids using only MSCs.^[^ ^149^ ^]^ Human cord blood MSC (hCBMSC)‐derived spheroids were grown and transplanted into mouse ischemic tissue. The hCBMSC spheroids were evaluated for apoptotic signaling, angiogenesis‐related signal pathways, and blood vessel formation both in vitro and in vivo. As expected, cell survival was higher in spheroids as compared to cells in monolayer culture. The spheroids improved viability of the transplanted cells and promoted angiogenesis, as evident by an increase in the number of microvessels within the spheroids.^[^ ^149^ ^]^ Similarly, when *β*‐cell pseudoislets were cocultured with MSCs, they exhibited insulin‐producing phenotype and secreted angiogenic and antiapoptotic proteins.^[^ ^141^, ^150^ ^]^ Both reports demonstrated that MSC‐incorporated spheroids had enhanced viability, paracrine secretion, and vascularization after transplantation.

**Figure 6:**
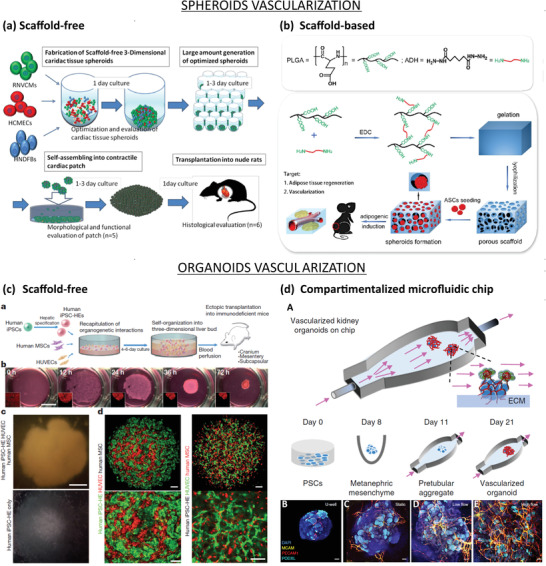
Vascularization approaches for spheroids (top) and organoids (bottom). a) Scaffold‐free approach to vascularize spheroids. RNVCMs, HCMECs, and hNDFs were cocultured at optimal cell ratios (70%:15%:15%) and plated into ultralow attachment 96 U‐well plates to form cardiac tissue spheroids. Then, the spheroids were collected and plated in low‐attachment dishes, allowing them to self‐organize into cardiac patch grafts under static conditions. Finally, the cardiac patch grafts were transplanted on the anterior wall of the left ventricle of arhythmic rats to induce spontaneous vascularization. Reproduced with permission.^[^ ^148^ ^]^ Copyright 2016, Elsevier Inc. b) Scaffold‐based approach to vascularize spheroids. PLGA activated by 1‐ethyl‐3‐(3‐dimethylaminopropyl) carbodiimide hydrochloride (EDC) and crosslinked with adipic dihydrazide, followed by lyophilization forms porous hydrogel. Seeding of ASCs onto hydrophilic surface induced cell aggregations, which resulted in ASC‐spheroids. Then, the spheroids were transplanted in the dorsum of nude mice to induce spontaneous vascularization. Reproduced with permission.^[^ ^147^ ^]^ Copyright 2017, Elsevier Inc. c) Scaffold‐free approach to vascularize organoids: a) Schematic representation of the paper's strategy: hiPSCs, hMSCs, and HUVECs cocultured on Matrigel to form liver organoids, which were transplanted into mice to induce spontaneous vascularization. b) Observation of cells in coculture overtime. Organoids formed within 72 h. c) Observation of hiPSC‐organoids (top panel) and conventional 2D cultures (bottom panel). Scale bar = 1 mm. d) Confocal images showing the presence of hiPSC‐derived hepatic endoderm cells (green) and HUVECs (red) inside liver organoids (left panel), or HUVECs (green) and hMSCs (red) inside hiPSC‐derived organoids. Scale bar = 100 µm. Adapted with permission.^[^ ^117^ ^]^ Copyright 2013, Springer Nature. d) Compartmentalized microfluidic‐based hybrid strategy: A) Kidney organoids were cultured in ECM substrate housed inside a perfusable millifluidic chip, subjected to controlled fluidic shear stress. B–E) Confocal 3D observations showing vascular markers in whole‐mount organoids, cultured under static U‐well, static, low‐FSS, and high‐FSS conditions. Scale bars = 100 µm. Adapted with permission.^[^ ^137^ ^]^ Copyright 2019, Springer Nature.

Coculture of EC‐incorporated spheroids with fibroblasts can also enhance vascularization. Fibroblasts are essential for production precursors for the ECM and therefore, it contributes to the stabilization of the newly formed vessel‐like structure.^[^ ^151^ ^]^ Noguchi et al. developed cardiac tissue spheroids by coculturing rat neonatal ventricular cardiomyocytes (RNVCMs), human cardiac microvascular endothelial cells (HCMECs), and hNDFs (**Figure** **6**a). The spheroids were then fused into a patch‐like construct and transplanted into rat hearts. Results showed that microvascular networks formed inside the spheroids, both in vitro and in vivo experiments.^[^ ^148^ ^]^

##### Vascularization Strategies of Spheroids: Scaffold‐Based Approach

Biomaterial‐based scaffolds have also been adopted for vascularization of spheroids as instructive guides to improve spheroid function and promote angiogenesis. In one study, adipose‐derived stem cells (ASCs) spheroids were covered with hyaluronan (HA) gel and chitosan–hyaluronan (CSHA) membrane and seeded onto the wound area on the dorsal skin of Sprague‐Dawley male rats. In vitro analysis demonstrated that ASC spheroids had higher gene expression of chemokines and cytokines when cultured on HA gel and CSHA membrane, suggesting an improvement in paracrine effects. Following transplantation, spheroids were observed near microvessels in the healing region of the skin. The enhanced paracrine effects upregulate angiogenic factors secretion, thereby stimulating angiogenic and wound healing processes.^[^ ^152^ ^]^

In another approach, MSC spheroids were entrapped within Arginine‐Glycine‐Aspartate (RGD)‐modified alginate hydrogels and transplanted into the dorsum of immunodeficient mice for 8 weeks. In vitro analysis demonstrated that these spheroids underwent osteogenic differentiation and exhibited enhanced VEGF secretion and reduced apoptosis. Furthermore, explants of hydrogels containing spheroids demonstrated improved osteogenesis in vivo.^[^ ^146^ ^]^

Human ASCs were used to generate spheroids, which were then seeded into dried porous poly(lactic‐*co*‐glycolic acid) (PLGA) scaffolds. The resulting constructs exhibited improved vascularization and adipogenic differentiation upon transplantation.^[^ ^147^ ^]^ Similarly, ASC spheroids in porous polyurethane scaffolds demonstrated enhanced angiogenic potential, as evidenced by greater microvessel density.^[^ ^153^ ^]^ In another study, hMSC/HUVEC spheroids seeded onto poly(propylene fumarate)/fibrin scaffolds showed enhanced vascular network formation.^[^ ^154^ ^]^

##### Vascularization of Organoids: Scaffold‐Free Approach

Prevascularized organoids have been transplanted into kidney,^[^ ^118^, ^119^ ^]^ brain,^[^ ^116^, ^155^, ^156^ ^]^ and liver.^[^ ^117^ ^]^ Among the various labs which attempt to form vascularized organoids, the most notable is the work of Takebe's group. They have successfully fabricated complex vascularized organ buds for kidney, heart, lung, brain, intestine, and pancreas using murine PSC‐derived progenitors, HUVECs, and MSCs.^[^ ^126^, ^157^ ^]^

Watson generated human intestinal organoids using hESCs or hiPSCs and transplanted them in the kidney capsule of immunocompromised mice.^[^ ^119^ ^]^ The grafted organoids were vascularized by the host vasculature and resembled the native human intestine with crypt‐villus architecture and underlying laminated submucosal layers. Cross‐section of the transplanted organoids, which showed mucous‐filled lumens and sheets of villi with capillary network, further indicated vascularization and good engraftment of organoids into the host kidney. The in vivo tissue was more differentiated and matured over time compared to in vitro tissue prior to transplantation.^[^ ^119^ ^]^ Similarly, spontaneous vascularization upon transplantation was also achieved for kidney organoids. Using the ALI method, van den Berg et al. generated kidney organoids from podocytes and grafted them into the renal capsule of immunocompromised mice for 28 days. The organoids developed in vitro anatomical‐like structures resembling a nephron including the glomerulus, the distal and proximal tubes, and the collecting duct. However, the in vitro tissue did not form a vascular network, probably due to the limited VEGF production of podocytes and the absence of ECs during in vitro development. Upon transplantation to a highly vascularized site, the organoids grew in size, differentiated progressively into mature kidney tissue, and developed their own vascular network that connected to the mouse vasculature, which supplied blood to their core.^[^ ^118^ ^]^

Stem cells can be codifferentiated into organ‐specific structures and ECs to obtain vascularized organoids and hESCs or hiPSCs have been successfully used to form cerebral organoids and ECs by codifferentiation.^[^ ^116^, ^155^, ^156^ ^]^ In this case, different protocols and culture conditions have been developed by different research groups and all studies showed organoids which formed tubular structures and perfused vascular networks in vitro. In Ham and Pham protocols, hESCs or hiPSCs were induced into neuroectoderms which were then introduced in cerebral organoids media and VEGF‐supplemented cerebral organoids media for organoid and endothelial differentiation, respectively. Alternatively, Cakir et al. induced the expression of ETV2, a transcription factor contributing to vessel development, to differentiate hiPSCs into ECs.^[^ ^116^ ^]^ Moreover, they reported their organoids could promote neuronal maturation and development of vascular networks with BBB characteristics. Thus, the preformed functional vessels eventually anastomosed with the host vasculature upon transplantation while the organoids generated without ECs did not survive after 2 weeks of transplantation.^[^ ^116^, ^155^ ^]^ All results strongly suggest the presence of endothelial cells is highly essential for proper vascularization and engraftment of organoids prior to transplantation.

Along with ECs, MSCs are also included in coculture experiments for vascularization due to their angiogenesis properties. When liver cells were cocultured with HUVECs and MSCs to form liver buds, the resulting 3D structures had liver‐specific functions, developed vascular networks and integrated with the host transplantation sites (Figure 6c).^[^ ^125^, ^158^ ^]^ Beside the liver, Takebe's group has also successfully developed complex vascularized organ buds for kidney, heart, lung, brain, intestine, and pancreas through self‐condensation procedures using murine PSC‐derived progenitors, HUVECs, and MSCs following implantation in host mice.^[^ ^126^ ^]^

**Table** **4** summarizes the significant case studies for the 3D cell culture vascularization strategies, cited or discussed in the text.

**Table 4:** Summary of case studies for 3D cell culture vascularization strategies

Vascularization method		Tissue/organ model	Biomaterial composition	Cellular composition	Duration of in vitro study	In vivo evaluation	Refs.
Spheroids	Scaffold‐ free	Bone	–	Osteoblasts, dermal microvascular ECs, normal dermal fibroblasts (all from human)	3 days	2 weeks	^[^ ^139^ ^]^
		Dental pulp	–	Dental pulp stem cells, HUVECs	3 days	4 weeks	^[^ ^129^ ^]^
		Liver	–	Rat hepatocytes, HUVECs	25 days	No	^[^ ^140^ ^]^
		Heart	–	Rat neonatal ventricular cardiomyocytes, human dermal fibroblasts, human CMECs	7 days	7 days	^[^ ^148^ ^]^
		BBB	–	Brain ECs, pericytes, astrocytes (all from primary human source)	3 days	No	^[^ ^128^ ^]^
	Scaffold‐based	Skin	Hyaluronan; chitosan	ASCs	3 days	8 days	^[^ ^152^ ^]^
		Adipose tissue	PLGA hydrogel	ASCs	2–3 weeks	3 months	^[^ ^147^ ^]^
		Bone	RGD‐modified alginate gel Polyurethane Poly(propylene fumarate); fibrin	MSCs ASCs hMSCs, HUVECs	2–3 days 3 days 1–3 weeks	2 months 2 weeks 9 days	^[^ ^146^, ^153^, ^154^ ^]^
Organoids	Scaffold‐free	Brain/BBB	–	hiPSCs	Up to 4 months	No	^[^ ^156^ ^]^
		Intestine	–	hESCs/hiPSCs	35 days	6 weeks	^[^ ^119^ ^]^
		Kidney	–	hPSCs	25 days	28 days	^[^ ^118^ ^]^
		Brain	– –	hESCs → ECs and organoids hiPSCs → ECs and organoids	Up to 4 months 54 days	30 days 7 days	^[^ ^116^, ^155^ ^]^
		Liver	–	hiPSC‐endoderm cells, hiPSC‐ECs, MSCs	3 days	14 days	^[^ ^125^, ^157^ ^]^
		Pancreatic islet, brain, heart, lung, intestine, kidney, liver fragments	–	Tissue fragment, HUVECs, hMSCs	1 day	1 month	^[^ ^126^ ^]^

#### 4.2.3 Limitations of Vascularized 3D Cell Culture Models

Both spheroids and organoids have great potential as vascularized models for disease modelling and drug development purposes. While they bring about promising outlook for the biomedical field, several limitations remain. First of all, both spheroids and organoids generation need a large number of cells to obtain a substantial quantity of tissue. Second, cellular microenvironment is the key factor to achieve viable and functional 3D structures with in vivo characteristics, while at the same time promoting angiogenesis.^[^ ^159^ ^]^ Therefore, ECM or a similar matrix, such as Matrigel, which is a complex protein mixture from mouse, is commonly used, mainly for organoids. However, due to the heterogeneous composition and immunogenic potential of currently used matrices, an alternative ECM‐mimicking source should be considered.^[^ ^13^ ^]^ Alternatively, biomaterial‐based 3D scaffolds have been employed to mimic the components of the ECM while providing structural support and external cues to guide cell–cell and cell–matrix interactions, leading to functional and vascularized spheroids.^[^ ^147^, ^152^, ^153^, ^154^ ^]^ While these scaffolds can provide mechanical and biochemical cues for cell growth within the 3D structures, lack of access to adequate supply of oxygen and nutrients to the center of the structure often results to necrotic core and premature growth in the outer layer of organoids, when missing an adequate vascularization of the 3D constructs.^[^ ^159^ ^]^

The key requirement for vascularization concerns the surrounding microenvironment, which has to support both angiogenesis and organoid formation.^[^ ^159^ ^]^ The incorporation of ECs in the cell culture can alleviate this problem by inducing in vitro prevascularization, leading to the formation of functional tubular vessels. This increase access to oxygen and nutrients, thanks to functional vessels, promotes cells survival, maturation, and differentiation to specific tissue. HUVEC‐covered hepatocyte spheroids had improved cell viability and liver‐specific functions such as increased albumin secretion and ammonia removal rates.^[^ ^140^ ^]^ Cerebral organoids generated from hPSCs formed tubular vessels with pericyte‐like cells wrapping around them, while promoting neural differentiation.^[^ ^156^ ^]^

As these techniques work with cocultures, factors such as cell ratios, seeding density, appropriate cell culture medium, and coculture time must be optimized. For example, while it was possible to form spheroids composed of human adipose‐derived mesenchymal stromal cells (hASCs) and HUVECs, vascular structures were only observed when 20% ASCs were cultured with 80% HUVECs in a 1:1 mixture of endothelial and adipogenic medium.^[^ ^160^ ^]^ Similarly, Noguchi's work showed that contracting vascularized cardiac spheroids were obtained by maintaining the following cell mixture: 70% CMs, 15% ECs, and 15% FBS.^[^ ^148^ ^]^

Despite their ability to nourish spheroids/organoids, preformed vessels need to be transplanted in a highly vascularized region to achieve optimal perfusion. The need to experiment on animal models poses a paradox since the one of the main goals of using 3D cell culture models is to reduce animal use in research. Nevertheless, vascularized brain organoids raise ethical concerns and call for consciousness assessment of animal models used in these experiments.^[^ ^161^ ^]^

Furthermore, the combination of spheroids/organoids platform with 3D bioprinting and microfluidic technology are necessary to achieve more comprehensive vascularized, physiologically relevant 3D models.^[^ ^134^, ^137^, ^162^ ^]^ A more in‐depth discussion on this topic is presented in Section 4.4.

### 4.3 3D Bioprinted Vascularized Models

In the last decades, the word biofabrication has been widely used in the scientific community to describe a plethora of processes aimed to manufacture complex products with a biologically relevant function built from biological building blocks, such as biomaterials, cells, or molecules.^[^ ^163^, ^164^, ^165^, ^166^ ^]^ Although biofabrication techniques for tissue engineering and regenerative medicine have been commonly classified into top‐down and bottom‐up,^[^ ^167^, ^168^, ^169^ ^]^ we adopt here the classification proposed by Groll et al.^[^ ^170^ ^]^ Considering the fabrication unit, two approaches can be distinguished, namely, bioprinting and bioassembly. While bioprinting uses molecules, that are assembled by means of additive manufacturing techniques based on computer‐aided design (CAD) models, bioassembly uses prefabricated cellular building blocks that can be automatically assembled. Both strategies are followed by a tissue remodeling and maturation phase, which is an integral part of the biofabrication process.^[^ ^169^ ^]^ Though some bioassembly strategies have achieved successful applications in vascularized tissue models (Section 4.3.4), bioprinting represents nowadays the cutting‐edge biofabrication technology in the field and will be the main focus of this section.

#### 4.3.1 Current Bioprinting Technologies

**Figure 7:**
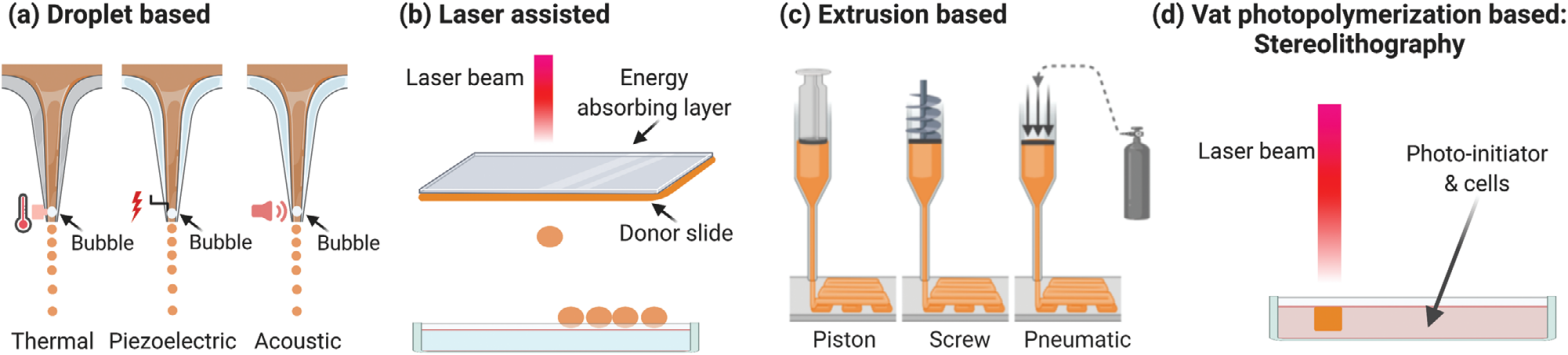
Schematic of bioprinting methods. a) Inkjet‐based bioprinting involves the formation of droplets of bioink by generating bubbles in the tip of the printer through thermal, piezoelectric, or acoustic energy. b) Laser‐assisted bioprinting is also based on the generation of droplets of bioink by the incidence of a laser beam on an energy absorbing layer coupled with a donor slide constituted of bioink. The droplets are then recovered on a dedicated platform. c) Extrusion is the most commonly used method; the ink is pressed through the nozzle either with a piston, a screw, or using pneumatic pressure. d) Vat photopolymerization requires the presence of a photoinitiator to cure the polymer loaded with cells. Created with Biorender.com.

Although the concept of 3D printing encompasses different technologies, as summarized in **Figure** **7**, most of them show common advantages for the vascularization of biomaterials: 1) the possibility to print vessels of different diameters, ranging from microvessels to vessels in the mm range, that can be surgically anastomosed; 2) the use of bioinks, whose composition can improve vascularization; 3) the ability to control the spatial arrangement of cells to promote the formation of vessel networks, eventually with branched, complex geometries.^[^ ^109^ ^]^ We provide here a general overview of the current 3D bioprinting technologies employed to vascularize tissue constructs. Later in this section, we summarize the definitions adopted and the critical bioprinting parameters. A more detailed description of the most used commercial bioprinters can be found, for instance, in the work by Ozbolat et al.^[^ ^171^ ^]^

##### Inkjet‐Based

This technology can be applied in a continuous mode or in a drop on demand mode. In the first case the printing ink needs to be electroconductive, which limits its application for biological purposes. Besides, the drop on demand mode is based on the deposition of droplets on the printing surface. To generate and eject the drops, thermal, piezoelectric, or acoustic approaches are used (Figure 7a). Thermal printing heads heat the bioink locally creating a bubble that pushes the drops through the nozzle. In the case of piezoelectric and acoustic actuators, vibration is at the origin of drop deposition. Compared to other printing techniques, inkjet bioprinting is low‐cost and allows for fast printing with high resolution (50 µm). This is a suitable technology for low viscosity bioinks (<10 mPa s) with a low cell density. Cell viability has been reported in the range of 80–95% using this method, due to the temperature and the mechanical stress.^[^ ^172^, ^173^ ^]^

##### Laser‐Assisted Bioprinting (LAB)

This technology, also known as laser‐induced forward transfer, is a drop on demand method based on the incidence of a pulsed laser beam on top of a donor slide in contact with an energy‐absorbing layer. When a bioink is placed next to the energy‐absorbing layer, a shockwave appears forming a jet of the bioink that is deposited as a drop on a collector slide (Figure 7b). High resolution (5–10 µm) and the possibility to work with a wide range of densities (1–300 mPa s) and to print the cells on solid or liquid substrates are the main advantages of this strategy. Other benefits are automation, reproducibility, and high throughput. Nevertheless, it is a very expensive technology that might cause cell damage. Other drawbacks are cell sedimentation and, when printing 3D constructs, the risk that working wavelengths alter cell organization.

##### Extrusion‐Based

The most popular 3D bioprinting technologies nowadays are pressure‐assisted, which are well adapted for highly viscous and, ideally, structurally stable solutions to avoid loss of shape (Figure 7c). For this purpose, most approaches in the literature combine bioprinting of the ink with in situ crosslinking after injection.^[^ ^109^ ^]^ It is also frequent to work at controlled temperature to assure good viscosity of the bioink and to induce in situ gelation. A drawback is that reproducibility depends on numerous parameters, namely, needle diameter, air pressure, speed of printing, temperature, and humidity. Assuring homogeneity of the bioink during the printing process is particularly relevant in cell‐loaded bioinks. Also, excessive pressure can result in cell damage caused by shear stress. Other disadvantages are low speed, low resolution, and clogging.

There are several commercially available 3D bioprinters, but the simplicity of the technology leads many research laboratories to manufacture customized printer based on their needs. To print different bioinks without crosscontamination, the use of multiple injectors is often adopted. In the case of vascularized materials, the use of coaxial needles is particularly advantageous to print tubular structures as will be seen in Section 4.3.2.

##### Vat Photopolymerization‐Based

The possibility to photocure polymers loaded with biomolecules and/or cells has open new perspectives to create tissue constructs. The process is based on a laser beam that irradiates a resin composed of a solvent, a photoinitiator and a polymer. The photoinitiator reacts to the light source releasing radicals or cations that start the polymerization of the resin. This technology was first applied to 3D print low cell compatible resins in the presence of photoinitiators, which were however highly cytotoxic. In the last years, the development of new photoinitiators has expanded the application of this technique to the biomedical field.^[^ ^174^ ^]^ The high precision and resolution, together with the possibility to incorporate photoabsorbers to prevent photopolymerization in defined regions, makes this technology particularly interesting to engineer vasculature.^[^ ^175^ ^]^ The extraordinary freedom of design to pattern highly complex hollow vascular‐like structures within biomaterials has been recently demonstrated.^[^ ^176^ ^]^

Another advantage of vat photopolymerization compared to extrusion is the possibility to use low viscosity resins that improve the resolution compared to high viscosity ones but that can lead to cell sedimentation. The major drawback of the technology is the cell damage caused by the laser and by oxidative stress due to the activation of the photoinitiators. Laser sources in the UVA–visible spectrum are preferred since they are less toxic than shorter wavelengths in the UVB and UVC regions. In the field of vascularization, the most used photoinitiators are Irgacure 2959 (maximum efficiency wavelength 275 nm) and lithium phenyl‐2,4,6‐trimethylbenzoylphosphinate (LAP, maximum efficiency wavelength 375 nm), the latter being the less cytotoxic one.^[^ ^174^, ^177^ ^]^

Depending on the light source to cure the polymers, vat photopolymerization can be classified in stereolithography (SLA) (polymer cured with a laser), digital light processing (DLP) (polymer cured with a projector), and continuous digital light processing (CDLP)/continuous liquid light processing (CLIP) (polymer cured with oxygen and light emitting diodes (LEDs)).^[^ ^174^, ^178^ ^]^

##### Definitions and Relevant Parameters in Bioprinting

Definitions are given to differentiate between cell‐loaded bioinks, hereinafter “bioinks,” and acellular bioinks that will be named “biomaterial bioinks,” according to Groll et al.^[^ ^179^ ^]^ Most of bioinks are composed of one or several materials, other than cells, being the number of studies using a material‐free approach very small, as described in Section 4.3.2. Biomaterial bioinks are generally printed to form a scaffold where cells are seeded in a following step, being the risk of heterogenous cell distribution greater, compared to cellular bioinks. In both cases, biomolecules can be incorporated in the ink to exert a biological effect on cells. Other nonbiological materials can also be added to affect cell function via mechanical or electrical cues. Materials can also act as mere supports during the printing process, or as sacrificial inks that are removed after the printing process.

Solution viscosity is one of the critical material parameters for inkjet or extrusion bioprinting. The degree of viscosity must permit smooth nozzle extrusion, with homogeneous texture during the whole printing process, and fast solidification after printing.^[^ ^173^ ^]^ Clogging of the nozzle is frequent due to excessive viscosity or to progressive cell sedimentation. When the solution is not viscous enough, the printed construct risks to collapse or to eventually lose its shape. Viscosity is therefore related to the printability of the material, or coprintability of several biomaterials, which must have shear‐thinning or thixotropic rheological behavior during the printing. To modify the solution, viscosity, concentration,^[^ ^180^ ^]^ or temperature^[^ ^181^ ^]^ can be tuned. Shao et al. used Gel/gelatin methacryloyl (GelMA) solutions cooled at −20 °C for 5 min to form a prebioink, which was then printed on a platform at 2 °C.^[^ ^181^ ^]^ Additionally, the syringe was turned over every 20 s to homogenize cell suspension. A similar approach was followed by Jin et al. by using a mixture of gelatin and alginate.^[^ ^182^ ^]^ For thermal sensitive materials, the printability can be improved by including sacrificial polymers in the bioink solution. Maiullari et al. mixed alginate with polyethylene glycol (PEG)–fibrinogen, followed by a curing step of the PEG–fibrinogen with UV and the final removal of the alginate with ethylenediaminetetraacetic acid (EDTA).^[^ ^183^ ^]^ Besides printability, viscosity can be also modulated to obtain complex geometries particularly relevant for vascularization. In an elegant work, Lin et al. 2019 reported how by increasing the viscosity of a sacrificial bioink made of Pluronic F127, it was possible to avoid viscous fingering at the interface between the printed features and the surrounding material to obtain smooth curved channels.^[^ ^75^ ^]^ The best way to evaluate viscosity and printability is to perform rheological studies to establish the optimal working ranges of viscosity and storage moduli (for an extensive review about printability and rheological characterization, the reader is referred to ref. ^[^ ^184^ ^]^). Ideal reported values of viscosity are 10 mPa s for droplet‐based bioprinting,^[^ ^173^ ^]^ with an upper limit of about 100 mPa s,^[^ ^185^ ^]^ 1–300 mPa s for LAB, and 30 to 6 × 10^7^ mPa s for extrusion.^[^ ^173^ ^]^

The diameter of the printed element also affects important physical properties of the final construct, such as porosity, mechanical strength, and height of the scaffold.^[^ ^173^ ^]^ In the case of extrusion, this parameter is closely linked to the needle/nozzle diameter, the printing pressure and speed, or the flow rate of injection. Low resolution of the extrusion technique remains one of the main limitations to properly vascularize materials by bioprinting and the formation of tubular structures with a diameter similar to small venules, arterioles, and capillaries still represents a challenge. Nozzle‐free strategies can represent an alternative due to better resolution, compatible with vessels below 100 µm, and less limitation in terms of viscosity and potential cellular toxicity.

Finally, when establishing bioprinting parameters, in addition to the aforementioned, it should not be forgotten that they all affect cell behavior and viability.

#### 4.3.2 Bioprinting Strategies for Vascularization

##### Sacrificial Bioprinting

Sacrificial bioprinting uses a biomaterial bioink whose sol–gel transition or gelation can be easily controlled. First studies used organic materials soluble in organic solvents,^[^ ^186^, ^187^ ^]^ and therefore incompatible with the incorporation of cells. Based on previous work where cotton candy was used as sacrificial material to form channels within PDMS,^[^ ^188^ ^]^ Miller et al. reported in 2012 the use of a carbohydrate mixture optimized for bioprinting and subsequent dissolution of interconnected and branched filaments with several diameters.^[^ ^98^ ^]^ The properties of the printed filaments allowed the formation of microchannels within a wide variety of cell‐loaded materials such as agarose, alginate, photopolymerizable PEG, fibrin, or Matrigel. This work inspired numerous studies using the same fugitive ink strategy to mimic the microvascular architecture.^[^ ^75^, ^95^, ^180^, ^181^, ^189^, ^190^, ^191^, ^192^, ^193^, ^194^ ^]^

**Figure 8:**
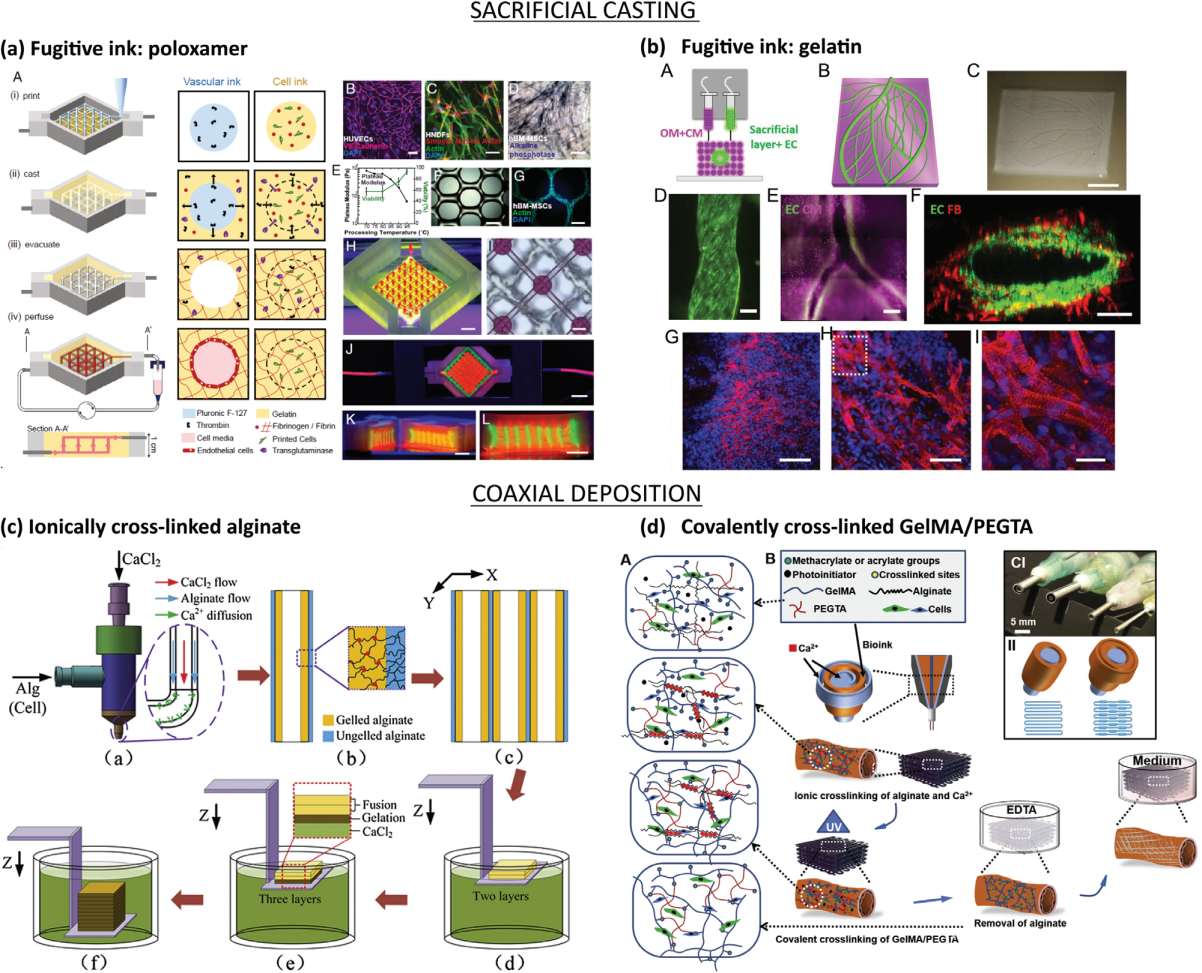
Bioprinting‐based vascularization strategies: sacrificial casting (top) and coaxial deposition (bottom). a) Bioprinting of thick vascularized tissues with sacrificial poloxamer: A) Manufacturing process in four steps: i) printing of the sacrificial poloxamer‐thrombin biomaterial bioink and of cell‐laden gelating bioink with endothelial cells; ii) casting of the gelatin/fibrinogen/transglutaminase that interacts with the thrombin diffused from the printed biomaterial causing gelification; iii) removal of the poloxamer by cooling down leading to empty channels; iv) perfusion of the channels with cell media that results in endothelialization of the channels. Three cell types were incorporated: B) HUVECs, C) hNDFs, and D) hMSCs. (Scale bar: 50 µm.) E) Cell viability and mechanical properties of the construct are affected by gelatin preprocessing temperature. hMSC‐laden bioink F) immediately after printing and G) after 3 days. H–K) Images of the bioconstruct. H) Sacrificial bioink colored in red and cell‐laden bioink in green. (Scale bar: 2 mm.) I) Bright‐field image from top. (Scale bar: 50 µm.) J) Construct in a perfusion chamber and K,L) cross‐sections. (Scale bar: 5 mm.) Reproduced with permission.^[^ ^95^ ^]^ Copyright 2016, PNAS. b) Bioprinting of thick cardiac patches with sacrificial gelatin. A) Two bioinks composed of decellularized omentum tissue (OM) + cardiomyocytes differentiated form iPSCs (CM) and sacrificial gelatin + endothelial cells (ECs). B) 3D‐ model of the cardiac patch. C) Printed cardiac patch. D–F) Fluorescence images of the printed cardiac patch with the ECs (green), CM (purple), and fibroblasts (red). (Scale bars: 100, 500 and 100 µm, respectively). The cardiac patch was implanted between two layes of the rat omentum and then explanted for analysis. G‐I) Fluorescence images of the explanted patch showing the sarcomeric actin of the CM in red and nuclei in blue. (Scale bars from left to right: 100, 50, 25 µm). Adapted with permission.^[^ ^190^ ^]^ Copyright 2019, WILEY‐VCH. Copyright 2019, The Authors. Published by Wiley‐VCH. c) Coaxial bioprinting of 3D hydrogels with microchannels using alginate: a) Schematics of the coaxial nozzle in which alginate and CaCl_2_ are co‐injected to form b) channels with an inner layer of ionically crosslinked alginate surrounded by ungelled alginate. c) Several channels are printed in parallel and then d) immersed in a bath with CaCl_2_ to promote e) gelation of the noncrosslinked alginate. f) This step is repeated several times to create a 3D construct. Reproduced with permission.^[^ ^193^ ^]^ Copyright 2015, Elsevier Inc. d) Multilayer coaxial bioprinting of perfusable 3D constructs with a blend bioink: A) The bioink gels through ionical crosslink of alginate with Ca^2+^ and photocrosslink of GelMA and polyethylene glycol (PEGMA) exposed to UV irradiation. B) Schematics of the coaxial nozzle in which the blend bioink is injected in between CaCl_2_ solution to cause immediate alginate gelation. After UV irradiation, the alginate is removed in contact with EDTA and the construct placed in cell culture medium. C‐I) Multilayered coaxial nozzles and II) schematics of the channel formation. Reproduced with permission.^[^ ^195^ ^]^ Copyright 2016, Elsevier Inc.

Poloxamers, also known as Pluronic, are poly(ethylene oxide)–poly(propylene oxide)–poly(ethylene oxide) (PEO–PPO–PEO) triblock polymers with a critical micelle temperature and concentration. This means that at low temperatures they are present in solution, whereas at high temperatures they form micelles and form a gel. In practice, some poloxamers, such as Pluronic‐F127, can be bioprinted at temperatures that do not compromise cell viability, and then at 4 °C they become liquid and can be washed, leaving a lumen where endothelial cells can be seeded. This approach has been used by the team of JA Lewis in combination with a fibrin casted gel, in several studies. In 2016, a preliminary study to form a proximal tubule model in a microfluidic chamber was published.^[^ ^191^ ^]^ Three years later, the same team optimized the composition of the Pluronic‐based fugitive ink, and succeeded to print a proximal tubule and a vascular channel that were seeded with epithelial cells and glomerular microvascular endothelial cells, respectively, under flow conditions.^[^ ^75^ ^]^ Also in 2016, they used the same kind of approach to combine HUVECs and hNDFs to form the vasculature, together with osteoinduced hMSC to form a microfluidic platform to create a relevant 3D model of bone (**Figure** **8**a).^[^ ^95^ ^]^ An originality in those works is how the authors made the printed vascular ink interact with the casted cell‐loaded hydrogel surrounding it. Briefly, the vascular ink contained thrombin, and the gel that was casted contained fibrinogen and transglutaminase. This way, thrombin diffused from the vascular ink to the surrounding gel causing crosslinking of the material. Using this strategy, the authors were able to form a thick (>1 cm) 3D chip with endothelialized channels that could be perfused with culture medium to differentiate hMSCs into osteogenic cells.

Gel is another material that is frequently proposed to form hollow microchannels. Two recent works have used Gel‐based fugitive inks to create relevant models of bone. In 2017, Khademhosseini's group reported the use of GelMA with a low degree of substitution to print cylinder rods of around 500 *μ*m within cylinder rods of photocrosslinked gelatin methacryloyl with a high degree of substitution and loaded with hMSC.^[^ ^192^ ^]^ After removal of the sacrificial ink, HUVECs were seeded in the central channel mimicking the architecture of long bones. Besides, Shao et al. have proposed direct coaxial bioprinting to form core–sheath fibers using Gelatin–GelMA, loaded with HUVECs and mouse osteoblast, respectively, in a single printing step at 2 °C (Figure 8c).^[^ ^181^, ^193^ ^]^ After photocuring GelMA, the temperature is set at 37 °C to liquefy gelatin. The construct is left under static culture conditions for 3 h, to allow HUVECs to adhere, and then dynamic cell culture is done using a shaker. In the same work, authors used this approach to seed HUVECs and human breast cancer cells (MDA‐MB‐231) to create a cancer model. This biofabrication method presents numerous advantages due to the ability to print complex shapes with controlled heterogenous composition, in a relatively fast way. Nevertheless, further research is needed to confirm the presence of an endothelialized and perfusable lumen.

The use of bioprinting to vascularize hepatic constructs is not yet widespread.^[^ ^180^, ^196^ ^]^ Recently, a preset extrusion bioprinting technique using alginate as sacrificial ink was employed for liver multiscale tissue engineering.^[^ ^180^ ^]^ A preset cartridge was prepared with collagen 3%, loaded with cells, and alginate 3% as fugitive material. The design was established to mimic the hepatic lobule, with EA.hy 926 endothelial cells around the lumen (150–200 µm), in the external surface of the construct and radially interconnecting both surfaces. In the space between ECs, hepatic cells (HepG2/C3A) were printed. Using a preset cartridge allows to control the spatial disposition of the cells with just one printing head. However, compared to other strategies, the dimensions of the printed construct are smaller (4 mm width × 5.2 mm height × 2.5 mm thick). Alginate was also chosen to prepare vascularized cardiac tissue (Figure 8b).^[^ ^194^ ^]^ The aim of this work was to prepare a tissue construct for personalized therapy and drug testing. For this purpose, authors used decellularized omentum (peritoneum) to form a thermoresponsive hydrogel to print CMs, and sacrificial alginate to bioprint HUVECs. In the cardiovascular field also, the work by Maiullari et al. describes the use of coaxial bioprinting to prepare a cardiac patch.^[^ ^183^ ^]^

##### Coaxial Deposition

Coaxial deposition systems use concentrical nozzles to i) crosslink the bioink during the extrusion process and ii) directly print tubular structures that can mimic the multilayered organization of the vasculature. In the mentioned work by Maiullari et al., a microfluidic printing head was used to perform coaxial microextrusion.^[^ ^183^ ^]^ The inner needle injected a bioink composed of alginate, PEG–fibrinogen and cells, either HUVECs or iPSC‐derived cardiomyocytes, whereas the external needle injected a CaCl_2_ solution to crosslink the alginate. After bioprinting, UV was applied to crosslink PEG–fibrinogen, and then alginate was removed by EDTA washing. Notably, the authors could engineer fibers with the two cell types in a “Janus” conformation that proved to be the most effective to generate vessel‐like structures, compared to alternating layers of cells at two different ratios.^[^ ^183^ ^]^

Another interesting example of coaxial printing is the work by the team of Khademhosseini, which used this technology to print perfusable tubular constructs with needles ranging from 14G to 30G leading to internal diameters ranging from about 400 µm to 1 mm (Figure 8d).^[^ ^195^ ^]^ As in previously mentioned works by the same group, GelMA with an adjusted degree of substitution was used together with alginate as sacrificial ink. During the printing process, alginate was ionically crosslinked with Ca^2+^. Once the GelMA was photocrosslinked, the construct was washed several times and treated with EDTA to remove all the cationic ions. To obtain a stable tubular construct after removal of the alginate and improve the mechanical properties of the GelMA after crosslinking, different amounts of polyethylene glycol tetra acrylate (PEGTA) were included in the mixture. This study was mainly focus on the biofabrication method to prepare endothelialized constructs, and the cells employed were HUVECs and MSCs. Soon after, they applied the coaxial extrusion technology to prepare an endothelialized myocardium and a heart‐on‐a‐chip.^[^ ^108^, ^197^ ^]^ In this case, plain microfibers with a diameter of 300 µm were printed leading to homogenous HUVECs distribution. Interestingly, the authors reported a progressive migration of the cells to the surface of the microfibers, as alginate was released. Though cells formed a monolayer similar to an endothelium after 15 days, the final constructs did not present a lumen and were not perfusable.

Pancreatic islets were printed together with endothelial progenitor cells (EPCs) using a coaxial extrusion nozzle for the treatment of type I diabetes.^[^ ^198^ ^]^ Similar to previous works, a mixture of alginate and GelMA was used for ionic crosslinking and photocrosslinking, respectively, but in this case the endothelial cells were printed around the fiber containing the islets. Unexpectedly, the presence of EPCs did not improve islets function. On the contrary, the authors reported reduced insulin secretion of the islets probably due to reduced diffusion of glucose and hypoxia in the core fibers.

In the work by Leucht et al., the authors printed two different compartments with two bioinks to engineer vascularized bone bioconstructs.^[^ ^199^ ^]^ By mixing Gel, GelMA, and acetylated gelatin methacryloyl (AcGelMA), the authors significantly reduced the stiffness of the native G while increasing the swellability. This bioink loaded with hDMECs was printed in a concentrical compartment next to a second compartment where human adipose‐derived stem cells (hADSCs) differentiated in osteoblasts were previously bioprinted. The transparent vascularization gels were cured using a LED‐UVA lamp (385 nm). The authors demonstrated that softer materials led to better results in terms of number of vascular networks, length and number of nodes. Another way to print different bioinks or biomaterials bioinks, is to use multihead printers. In the work by Jang et al., three different bioinks loaded with human cardiac progenitor cells (hCPC) or hMSC, or a mixture of both were printed to fabricate cell patches for cardiac repair.^[^ ^200^ ^]^ They used decellularized ECM as biomaterial, with vitamin B_2_ and VEGF to improve vascularization, and implanted the construct in a rat model of heart ischemia. Results demonstrated the benefits of a patch with a specific pattern of CPCs and MSCs, which improved cardiac function and reduction of fibrosis, together with an increased neovascularization.

The possibility to print several bioinks in the same construct was exploited to create a gradient of growth factors in a construct for bone vascularization.^[^ ^192^ ^]^ As described previously in this section,^[^ ^195^ ^]^ Gel was prepared with two degrees of substitution, low and high. The low GelMA was used as sacrificial biomaterial bioink to form a hollow channel of around 500 µm inside the construct to form a perfusable blood vessel, mimicking the architecture of long bones. Concentric rods with four different formulations were printed to create both vasculogenic and osteogenic niches. By modifying the GelMA composition (low to high), the cells ratio (HUVECs and hMSCs), the silica nanoplatelets, and VEGF concentrations, the authors engineered a perfused scaffold with gradients of biochemical cues to promote both osteogenic differentiation and vascularization. In contrast to the previously mentioned studies,^[^ ^195^, ^198^, ^201^ ^]^ in this case, crosslinking of GelMA occurred in the capillary, before extrusion of the bioink. Another bioactive compound that has been incorporated in a biomaterial bioink for bone tissue engineering is nanohydroxyapatite (nHA).^[^ ^202^ ^]^ In this work, a mixture of gelatin and nHA was printed using Pluronic as sacrificial support to allow the crosslinker genipin to act during 48 h. Then, Pluronic was removed and HUVECs, hMSCs, and/or osteodifferentiated hMSCs were added in a solution made of GelMA–fibrin, which was photocrosslinked.

##### Stereolithography

The photocuring of polymers to engineer tissue vasculature is still at its early stage. Even if works using this strategy to vascularize tissue relevant constructs are very few, they hold great promise in view of the rapid evolution of the technique. In 2017, Zhu et al. used this technique in a pioneer work to bioprint a model of liver including HUVECs, MSCs, and HepG2 cells.^[^ ^203^ ^]^ This construct was subcutaneously implanted in a murine model demonstrating the anastomosis of the implant. Miri et al., faced one of the main limitations of this technology by building up a microfluidic device to allow stereolithography of a multimaterial construct.^[^ ^204^ ^]^ This way, they produced a simplified model of breast cancer including HUVECs and MCF7 cancer cells. Another model of breast cancer using SLA was more recently developed by Cui et al. to evaluate migration of metastatic cells to bone.^[^ ^205^ ^]^

In an elegant work published in 2019 in Science, Grigoryan et al. proposed the incorporation of food additives as photoabsorbers to form hydrogels with very complex and intricated networks to mimic several tissues, including an alveolar model.^[^ ^176^ ^]^ They also created a prevascularized construct with a network of HUVECs connected to hepatocyte aggregates, which was subcutaneous implanted. Hepatic cells functionality two weeks after implantation was demonstrated but the benefits of including an endothelial cell network in the production of albumin was not proved, although histological examination evidenced the anastomosis of the implant.

Vat‐photopolymerization can be combined with other 3D‐printing techniques. This is the case of the recent work by Hann et al., in which fused deposition modeling (FDM) for sacrificial PVA printing was combined with SLA for GelMA and PEGDA curing to build a channeled construct as model of bone tissue.^[^ ^206^ ^]^ Compared to the use of photoabsorbers to form hollow channels, the resolution of FDM was however really low, leading to vessels of several hundreds of micrometers.

Bioprinting holds great potential in the fabrication of diseased tissues as well, even if studies in this regard are still limited.^[^ ^181^ ^]^ Besides the case studies already mentioned, Liu et al. have recently proposed a model of atopic dermatitis fabricated by hybrid biofabrication combining electrospinning and extrusion bioprinting for the study of this skin disease and drug testing.^[^ ^207^ ^]^ For a comprehensive review about hybrid biofabrication, we refer the reader to ref. ^[^ ^208^ ^]^.

##### Scaffold‐Free Bioprinting and Alternative Strategies

A promising bioprinting strategy for vascularization in alternative to scaffold bioprinting is scaffold‐free bioprinting, which is based on the capacity of cells to self‐assembles after bioprinting and spontaneously form constructs that mimic the native tissue architecture and function. However, this strategy requires a large number of cells as well as a postprinting incubation period that prolongs the process and increases the costs. This explains why the number of studies using this technique to recreate the vasculature is currently limited and mainly focused on the fabrication of larger blood vessels (≥1 mm).^[^ ^209^, ^210^, ^211^, ^212^ ^]^

All the works described in this section so far deal with extrusion bioprinting. There are however two examples of laser induced forward transfer worth mentioning within the scope of this review. In 2011, Gaebel et al. reported the fabrication of a cardiac patch using a polyester urethane urea patch immersed in Matrigel.^[^ ^213^ ^]^ Using laser bioprinting, HUVECs and hMSCs were printed on the patch following a defined 2D pattern. This patch was implanted in an infarcted rat model and improvement of some cardiac functions and neovascularization were observed. More recently, intraoperative bioprinting of stem cells from the apical papilla and HUVECs using LAB has been successfully done to treat a mouse calvaria defect.^[^ ^214^ ^]^ **Table** **5** summarizes significant case studies for the bioprinting vascularization strategies, cited or discussed in the text.

**Table 5:** Summary of case studies for bioprinting vascularization strategies. Abbreviations not used previously: Col: collagen; GMECs: glomerular microvascular endothelial cells; hFob: human fetal osteoblasts; hiPSC‐CM: induced pluripotent stem cell‐derived cardiomyocytes; hiPSC‐EC: induced pluripotent stem cell‐derived endothelial cells; I: inner diameter; LAP: lithium phenyl‐2,4,6‐trimethyl‐benzoyl‐phosphinate; O: outer diameter; PCL: polycaprolactone; PTECs: proximal tubule epithelial cells; SCAPs: stem cells from the apical papilla

Vascularization method	Coaxial	Sacrificial	Organ/tissue model	Needle diameter	Vessel caliber	Biomaterial composition	Cellular composition	Duration of in vitro study	In vivo evaluation	Refs.
Extrusion‐based	No	No	Bone	0.33 mm (I)	Microvessels	Gel, GelMA, Ac‐GelMA	hDMECs, hADSCs, hADSCs differentiated in osteoblasts	14 days	No	^[^ ^199^ ^]^
			Heart	26G	Microvessels	PCL, heart‐derived ECM	MSC, CPC	5 days	Yes	^[^ ^200^ ^]^
			Skin	0.25 mm	Microvessels	PLGA, fibrin	iPSC‐ECs, perycites, neonatal fibroblasts, keratynocytes	7 days	No	^[^ ^207^ ^]^
			Heart	1.6 mm	1 mm	Agarose, alginate, platelet rich plasma	HUVECs, H9c2 CM	14 days	No	^[^ ^190^ ^]^
			Liver	0.25 mm	Microvessels	PCL, Col	HUVECs, hLFs, hepatocytes	14 days	No	^[^ ^196^ ^]^
		Yes	Liver	0.5 mm	0.15–0.2 mm	Col 3%, alginate 3% (sacrificial)	EA.hy 926, HepG2/C3A	10 days	No	^[^ ^180^ ^]^
			Bone	0.7 mm	Microvessels	Gel/nHA, Gel‐MA/fibrin	HUVECs, hMSC hMSCs differentiated in osteoblasts	5 weeks	No	^[^ ^202^ ^]^
			Heart	30G	0.3–0.4 mm	Decellularized momentum, alginate (sacrificial)	hiPSC‐ECs, hiPSC‐CMs, HUVECs, rat CM, fibroblasts	7 days	No	^[^ ^194^ ^]^
			Bone	0.5 mm	0.5 mm	GelMA‐high, GelMA‐low (sacrificial)	HUVECs, hMSCs	21 days	No	^[^ ^192^ ^]^
			Bone	0.1–0.4 mm	0.4 mm	Gel, fibrinogen, thrombin transglutaminases, poloxamer (sacrificial)	HUVECs, hNDFs, hMSCs	>6 weeks	No	^[^ ^95^ ^]^
			Kidney	0.41 mm	0.2 mm	Gel, fibrinogen, transglutaminase, poloxamer (sacrificial)	GMECs, PTECs	18 days	No	^[^ ^75^ ^]^
	Yes	Yes	Cancer tissue and osteogenic tissue	27G (I), 17G (O)	0.2–1 mm	GelMA, Gel (sacrificial)	HUVECs, MDA‐MB‐231, MC3T3‐E1	20 days	No	^[^ ^181^ ^]^
			Heart	26G (I), 19G (O)	Microvessels	PEG, fibrinogen, alginate (sacrificial)	HUVECs, iPSC‐CMs	7 days	Yes	^[^ ^183^ ^]^
			Heart	27G (I), 18G (O)	0.2 mm	GelMA, Alginate (sacrificial)	HUVECs, neonatal CM	28 days	No	^[^ ^197^ ^]^
			–	27–30G (I), 18–25G (O)	0.3–1.5 mm	GelMA, PEGTA, alginate (sacrificial)	HUVECs, MSCs	21 days	No	^[^ ^195^ ^]^
			Cardiac	27G (I), 18G (O)	0.3 mm	GelMA, alginate (sacrificial)	HUVECs, neonatal CM	33 days	No	^[^ ^108^ ^]^
			Pancreas	0.4 mm	Microvessels	GelMA, alginate (sacrificial)	hEPCs, pancreatic islets (organoids)	15 days	Yes	^[^ ^198^ ^]^
Laser‐based	–	–	Heart	–	Microvasculature	Polyester urethane urea patch, Matrigel	HUVECs, hMSC	8 days	Yes	^[^ ^213^ ^]^
	–	–	Bone	–	Microvasculature	Col	HUVECs, SCAPs	–	Yes	^[^ ^214^ ^]^
Vat photopolymerization: SLA	–	–	Liver	–	Microvessels	Glycidal methacrylate‐HA, GelMA Photoinitiator: LAP	HUVECs, MSCs, HepG2	7 days	Yes	^[^ ^203^ ^]^
	–	–	Breast cancer	–	Microvessels	GelMA, PEGDA Photoinitiator: LAP	HUVECs, MCF7, C2C12, fibroblasts, MSCs	7 days	No	^[^ ^204^ ^]^
	–	–	Liver	–	Microvessels	GelMA, PEGDA Photoinitiator: LAP Photoabsorbers: tartrazine, curcumine, anthocyanine	HUVECs, hepatic aggregates (rat primary hepatocytes and NHDFs)	–	Yes	^[^ ^176^ ^]^
	–	–	Breast cancer	–	500 *μ*m and microvessels	GelMA, PEGDA Photoinitiator: Irgacure 2959	HUVECs, breast cancer cell lines: MDA‐MB‐231 and MCF‐7, hFob	14 days	No	^[^ ^205^ ^]^
Dual 3D printing (SLA and FDM)	–	–	Bone	–	0.5–1 mm and microvessels	GelMA, PEGDA, PVA (sacrificial) Photoinitiator: Irgacure 2959	HUVECs, hMSCs	20 days	No	^[^ ^206^ ^]^

#### 4.3.3 Limitations of Bioprinted Vascularized Models

3D bioprinting is an interesting technique for tissue engineering and particularly for vascularization but some current limitations still need to be addressed. As already mentioned, an important drawback concerns the poor resolution that currently makes extrusion printing of objects below 100–200 µm a real challenge. This limitation is even more important when it comes to direct channel printing by coaxial extrusion. That is why obtaining fully prevascularized constructs by bioprinting is not currently possible and the formation of microvasculature requires a postimpression maturation stage that can last several weeks. Other bioprinting techniques, such as LAB, show better resolution, but their use for tissue vascularization is currently limited, mainly due to high cost and limitations to print multiple materials.^[^ ^185^ ^]^

The homogeneity of the bioink during the bioprinting process, particularly relevant in the manufacture of larger constructs, represents another drawback. Cells at high concentrations tend to sediment, making the bioink not homogeneous. Moreover, the viscosity of the bioinks fundamental for its printability since it determines the cell density, it affects the mechanical properties of the final construct as well as the cellular viability and behavior (proliferation, differentiation, migration, etc.). Future studies should pay more attention to this aspect and carry out experiments that help to identify the optimal mechanical properties to promote adequate vascularization.^[^ ^215^ ^]^ In this regard, it is worth mentioning the extrusion bioprinting studies that are already being carried out in space, where microgravity allows the use of less viscous bioinks and the formation of particularly interesting geometries for vascularization, such as voids and tunnels.^[^ ^216^ ^]^

Finally, we have seen that a common strategy is the printing of photopolymerizable materials in the presence of a photoinitiator. These materials are often obtained by chemical modification of natural polymers, such as Gel, to incorporate methacrylate groups that polymerize after irradiation at a certain wavelength and in the presence of a photoinitiator. There are many studies focused on the development of cytocompatible photoinitiators, since those currently used are not considered totally harmless to the body and the presence of methacrylate groups can pose a problem for therapeutic use.^[^ ^217^ ^]^ Furthermore, as already mentioned, the presence of these groups creates materials with mechanical properties that should be further investigated.

Bioprinting is a relatively young technology that has come a long way in the last decade, opening up previously unthinkable possibilities for tissue engineering. Current limitations are mainly due to the bioprinting method and can be overcome by combining several printing strategies on a single platform.^[^ ^216^ ^]^ We envision that the advances of this technology over the next few years will contribute considerably to the development of vascularization strategies of physiologically relevant models.

#### 4.3.4 Bioassembly Strategies for Vascularization

##### Micromodule Assembly Strategies

Micromodule assembly refers to a category of modular TE strategies in which microscale building blocks are assembled to create larger tissues,^[^ ^218^ ^]^ with the advantage that the single units provide cells with efficient gas exchange and nutrients supply at the microscale and vascular networks can be easily integrated.^[^ ^219^ ^]^ The formation of modular vascular tubes is commonly achieved by using micromolds or by creating cell‐laden microgels, which are then assembled by photopolymerization,^[^ ^220^ ^]^ random packing,^[^ ^221^ ^]^ or direct assembly.^[^ ^222^ ^]^ Despite the scalability of these technologies, which provide dense cellular population while ensuring perfusion and diffusion and enable to control features at the microscale by tuning the building blocks properties, the lack of some fundamental requirements, as the mechanical stability, hampers their translation toward clinical application and successful engineering of vascularized tissue constructs.^[^ ^222^ ^]^

##### Cell Sheet Engineering

Scaffold‐based TE approaches are often limited to low cellular density, lack of a functional vascular network and, consequently, inability to create thick constructs that do not undergo necrosis.^[^ ^223^ ^]^ Cell sheet engineering has emerged in the 90's as scaffold‐free approach for the manufacturing of 3D cellular constructs with native tissue properties,^[^ ^218^ ^]^ and it has been successfully applied for cornea and trachea reconstruction, production of skin and bladder equivalents and myocardial tissue regeneration.^[^ ^224^, ^225^ ^]^ The technique consists of growing cells, that spontaneously produce ECM and form sheets, and subsequently assembling of the sheets by stacking or rolling them to obtain 3D or cylindrical tissue engineered blood vessels (TEBVs).^[^ ^226^, ^227^ ^]^ This technique has been used to engineer artificial vessels composed of up to three cellular layers (adventitia, media, and intima) that have been used as artery models and grafted in vivo to promote regeneration of the host vasculature.^[^ ^226^, ^228^ ^]^ Recently, the sheets manipulation has been improved by using temperature‐responsive culture substrata as poly(*N*‐isopropylacrylamide) (PIPAAm), that enable sheets release by simply lowering the temperature.^[^ ^227^ ^]^ Thick cardiac tissues (1 mm), prevascularized in vitro, were fabricated by multistep implantation of stacked sheets into animal models, that showed pulsatile cardiac tubes with beating up to 1 year and formation of microvasculature in vivo.^[^ ^223^ ^]^ Though cell sheet engineering is mainly used in therapeutics and regenerative medicine,^[^ ^229^ ^]^ the physiological tissue architecture and mechanical properties that can be achieved with this strategy make it interesting for developing highly organized and densely vascularized tissue models.

##### Nanofabrication

Most of the biofabrication techniques require a maturation phase of the tissue after assembly, usually carried out with bioreactors, which provide the tissue with nutrients, mechanical stimuli, and flow under dynamic culturing conditions.^[^ ^166^ ^]^ To overcome these limitations and provide cells with nanostructured scaffolds, nanotechnology‐based strategies have been used to fabricate tissues and vascular‐like structures:^[^ ^164^, ^230^ ^]^ phase separation and self‐assembly of peptidic domains of biological polymers, as collagen or elastin, have been used as strategies to engineer nanofibers, nanotubes and nanowires for vascular TE applications.^[^ ^231^, ^232^ ^]^ However, electrospinning is the main nanofabrication technique for vascularized constructs:^[^ ^230^, ^233^ ^]^ tubular scaffolds have been electrospun by using rotating mandrels or combination with electrospraying to create highly cellularized constructs,^[^ ^234^ ^]^ and multilayer core–shell constructs resembling the blood vessels structure have been manufactured by coaxial electrospinning.^[^ ^235^, ^236^, ^237^ ^]^ Electrospun scaffolds for vascular TE have been manufactured with a variety of natural and synthetic polymers and their combination in blends leads to devices with physiologically relevant mechanical behavior while promoting cell adhesion and proliferation.^[^ ^238^, ^239^, ^240^, ^241^ ^]^ The fibrous and porous architecture created by electrospinning mimics the in vivo ECM nanoenvironment and the fibers can be easily functionalized or grafted with molecules, peptides, drugs, or growth factors to promote cell adhesion, endothelialization, and antithrombogenic properties.^[^ ^242^, ^243^, ^244^ ^]^ However, few electrospun vascularized organ‐specific in vitro models have been reported,^[^ ^245^ ^]^ as most of the works use electrospun membranes or meshes for coculturing of cells with no physiologically relevant 3D vasculature.^[^ ^246^ ^]^ In fact, although electrospinning has been used for bone, skin, heart, liver, ligament, and kidney TE, it finds its main application in tissue repair and regeneration, as wound healing and dressing,^[^ ^247^ ^]^ osteochondral implants,^[^ ^248^, ^249^ ^]^ and tissue engineered vascular grafts (TEVGs).^[^ ^238^ ^]^ Moreover, it shows several limitations as i) low production rate,^[^ ^250^ ^]^ ii) pore size and fibers density that hinders cell infiltration,^[^ ^243^ ^]^ and iii) 2D thin shape at the macroscopic scale.^[^ ^250^ ^]^ Although some drawbacks have been addressed, for instance, cell infiltration can be increased by surface treatments or by coupling with other techniques to enhance macroporosity,^[^ ^251^ ^]^ and thick scaffolds can be engineered by multilayered electrospinning,^[^ ^252^ ^]^ bioprinting remains nowadays the most used and versatile technique for the biofabrication 3D vascularized tissue models.

### 4.4 Hybrid Strategies

**Figure 9:**
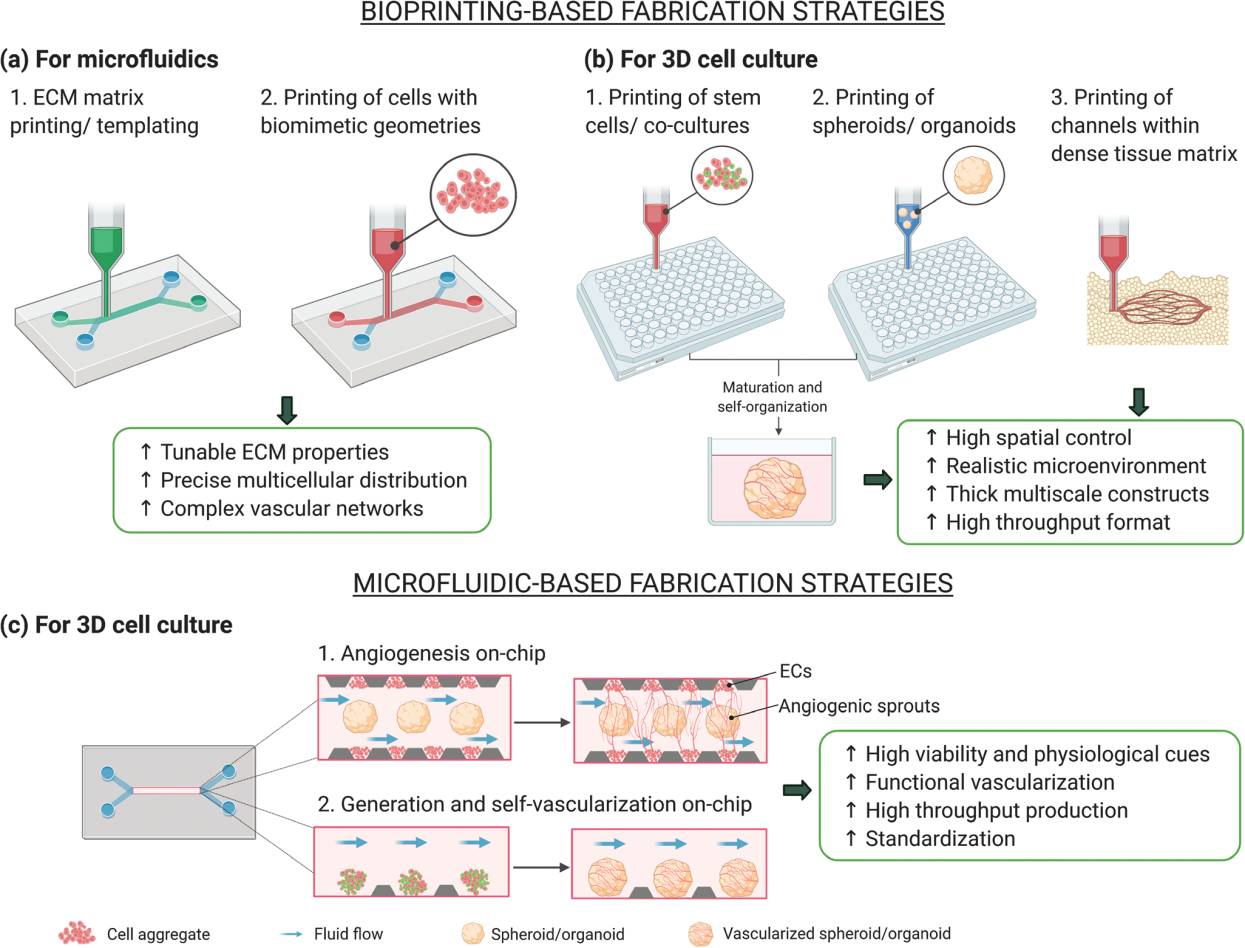
Hybrid strategies for vascularization. The hybrid approaches are divided into a,b) bioprinting‐based and c) microfluidic‐based. The main advantages of the application of these fabrication strategies for each model are shown in the green panels. Created with BioRender.com.

In the last years, the need to engineer sophisticated biomimetic in vitro models has led researchers to combine different vascularization techniques discussed so far in the same manufacturing process, making classification in distinct classes often reductive. The rise of hybrid strategies for vascularization has the advantage that the unique features and strengths of different fabrication strategies for vascularization of physiologically relevant 3D models can be recapitulated on a single platform and we report here some significant examples of this approach (**Figure** **9**).^[^ ^251^, ^252^ ^]^

#### 4.4.1 Bioprinting‐Based Hybrid Fabrication Strategies

Many studies have focused on the use of 3D bioprinting strategies, discussed in detail in Section 4.3, for the fabrication of vascularized organ‐on‐a‐chip platforms (Figure 3f). This approach shows several advantages such as the capability of recreating physiological‐like multicellular spatial organization within the device and direct manufacturing of 3D perfusable vascular geometries, reducing the fabrication steps and moving toward more reproducible and automated strategies.^[^ ^253^ ^]^ Moreover, this technique has shown its potential in vascularizing large tissue constructs and integrating patient‐derived cells, representing a valuable tool for personalized medicine.^[^ ^95^, ^254^, ^255^ ^]^ Bioprinting can be used either to i) print hydrogels as template for channels fabrication on‐chip or to ii) directly print vascular networks on‐chip from cell‐laden boinks (Figure 9a). These models, often embedded in an ECM matrix, are commonly perfused by integration within microfluidic bioreactors, produced by soft lithography or 3D printing technologies.^[^ ^19^, ^256^, ^257^, ^258^ ^]^

In a recent work, a perfusable liver model was fabricated with GelMA hydrogel loaded with hepatocytes by using agarose as fiber template.^[^ ^103^ ^]^ The cell‐laden matrix was casted in a poly(methyl methacrylate) (PMMA) mold and the agarose was bioprinted in the shape of a channel by microextrusion. After UV photocrosslinking of GelMA, the agarose fiber was removed to form the hollow channel and the device was embedded in a PDMS–PMMA bioreactor for perfusion. A functional lumen was obtained by subsequent seeding of HUVECs in the empty channel and the platform was used for drug toxicity assays. Lin et al. used extrusion‐based sacrificial bioprinting to engineer 3D vascularized proximal tubule models for the study of kidney reabsorption phenomena.^[^ ^75^ ^]^ They used Pluronic F127 and high‐molecular‐weight poly(ethyleneoxide) (PEO) as fugitive ink to print colocalized convoluted proximal tubule and vascular channel embedded in an ECM matrix of gelatin and fibrin (Figure 4d; see Section 4.3.2).^[^ ^191^ ^]^ A silicon gasket holding the structures allowed for perfusion of the tubule after dissolution of the fugitive ink at 4 °C. Proximal tubule epithelial cells and glomerular microvascular endothelial cells were seeded to form a functional epithelium and endothelium, respectively. Studies of albumin and inulin uptake confirmed selective reabsorption mechanism from the tubule to the vascular network and glucose reabsorption five‐ to tenfold higher compared to Transwell‐based models. The reabsorption functions of the tubule and the role of the endothelium were investigated as well after administration of glucose transport inhibiting drug and simulation of hyperglycemia conditions.

Although bioprinting techniques are increasingly used for printing perfusable microfluidic networks, the bioprinting step is often limited to the fabrication of polymeric tubular structures, which are successively washed to form hollow channels and seeded with cells, as described above.^[^ ^75^ ^]^ Recent works are focused on the bioprinting of cell laden gels on‐chip: this strategy allows a reduction of the fabrication time by eliminating the need for the cell seeding step and it ensures a more precise and homogeneous cellular distribution and alignment, eventually in complex multilayered geometries.^[^ ^104^, ^105^, ^259^ ^]^ In this context, coaxial needle technology has been used in several studies to directly fabricate endothelialized perfusable tissues.^[^ ^108^, ^195^ ^]^ 3D multilayer circumferential channels have been recently engineered by using single‐step coaxial needle manufacturing to reproduce human tubular tissues as urethra and blood vessels.^[^ ^104^ ^]^ A GelMA and alginate hydrogel blend combined with eight‐arm poly(ethylene glycol) acrylate with tripentaerythritol core (PEGOA) was used as bioink and extruded after cells encapsulation by using up to 3 circumferential needles. Urothelial tissue was created by bioprinting a core layer of human urothelial cells (HUCs) and an external layer of human bladder smooth muscle cells (HBdSMCs) while vascular tubular tissues were composed of HUVECs and hSMCs circumferential layers. Results confirmed long‐term viability (2 weeks), proliferation, and differentiation and showed the advantages of this method in creating functional tubular constructs for regenerative medicine and modeling (Table 2).

Recently, 4D bioprinting has emerged as technique for spatiotemporal control of networks self‐assembly by using smart materials that respond to external stimuli (temperature, pH, swelling, …). Thus, reversible self‐folding tubular constructs can be engineered and their properties controlled over time by tuning the external cues, making this approach particularly interesting for programming the cellular microenvironment and creating functional hybrid hierarchical bioconstructs.^[^ ^260^, ^261^ ^]^ Bioprinting strategies for vasculature and OOaC design have been extensively reviewed elsewhere.^[^ ^109^, ^110^, ^255^, ^261^, ^262^, ^263^ ^]^

3D bioprinting has been also used for fabrication of 3D cell cultures so as to overcome some of the current drawbacks, as spheroids/organoids variability and low throughput,^[^ ^159^, ^264^ ^]^ and spheroids/organoids models bioprinted on multiwell plates have been successfully developed for high throughput screening of compounds. Different bioprinting techniques have been adopted for either i) printing of PSC‐only bioinks, subsequently self‐organized in 3D aggregates, or ii) spheroid/organoid‐laden hydrogels (Figure 9b).^[^ ^265^, ^266^, ^267^, ^268^ ^]^ Using a commercial 3D bioprinter, Higgins et al. generated large numbers of homogeneous functional kidney organoids in an automated fashion. Organoids were bioprinted from hPCSs bioink into 96‐well plates and results showed formation of glomerular, epithelial and endothelial components and the capability to respond to drug‐induced toxicity. The bioprinter enabled the production of more than 600 organoids per hour while the manual generation was estimated to be about 30 organoids in the same timeframe.^[^ ^265^ ^]^ Vascularized adipose microtissues were created starting from a coculture of adipose‐derived stem cells and HUVECs spheroids.^[^ ^134^ ^]^ The spheroids were successfully used as bioprinting blocks encapsulated in a GelMA hydrogel mixed with a lithium‐based photoinitiator. The spheroid‐laden bioink was printed into a multilayer structure and the GelMA matrix was crosslinked through UVA irradiation. Results confirmed adipogenic differentiation, formation of vasculature and spheroids growth up to 14 days of culture. Vascularization of iPSC‐derived organ building blocks has been achieved via sacrificial writing into functional tissue (SWIFT) by the group of J Lewis.^[^ ^269^ ^]^ A matrix of collagen I and Matrigel was used as scaffold to tightly pack thousands of organoids that led to a highly dense tissue matrix after centrifugation. SWIFT was used for 3D printing of gelatin as sacrificial material within the matrix. After gelatin removal, the system could be perfused and functional lumens were formed by flow of HUVECs. This technique was used to generate perfusable cerebral organoids and cardiac spheroids and results confirmed the formation of functional tissue constructs with high cell density and in vivo‐like microarchitecture. Recently, complex tissues with relevant micro‐ and macroscale organization have been fabricated by bioprinting organoids building blocks within support hydrogels.^[^ ^268^ ^]^ The findings suggest the feasibility of engineering organoid‐based tissues at the centimeter scale, providing innovative functional constructs for regenerative medicine and drug research.

Bioprinted structures have also been used as delivery vehicles for organoids. Soltanian et al. 3D printed PLA tissue trapper containing collagen I and Matrigel for the transplantation of pancreatic organoids from human embryonic stem cells into the abdominal cavity of immunodeficient mice, observing anastomosis with the host vasculature and enhanced production of insulin thanks to the proper cell–cell and cell–matrix interactions.^[^ ^135^ ^]^

#### 4.4.2 Microfluidic‐Based Hybrid Fabrication Strategies

The use of microfluidics for the production and culture of organoids, also defined as organoid‐on‐a‐chip technology, is showing great potential in overcoming some of the main limitations of static 3D culture systems, as inefficient nutrients exchange, lack of standardization, and low throughput.^[^ ^124^, ^251^, ^270^ ^]^ Over the past years, microfluidic strategies have been used for generation of spheroids and organoids,^[^ ^271^ ^]^ in situ analysis and monitoring of organoids behavior,^[^ ^197^ ^]^ and to build automated platforms for drug screening and personalized medicine.^[^ ^272^, ^273^ ^]^

In the context of organoids vascularization, the two main microfluidic‐based approaches are i) direct generation on‐chip of the vascularized spheroid/organoid and ii) embedding of the spheroid/organoid and subsequent vascularization on‐chip (Figure 9c).^[^ ^137^, ^274^, ^275^, ^276^ ^]^ By using the first strategy, Jin et al. created vascularized liver organoids on‐chip. The liver organoids were composed of induced hepatic cells cocultured with HUVECs and they were embedded in a 3D decellularized liver ECM, used as scaffold. The system was integrated in a pump‐free microfluidic device under continuous flow. The encapsulation of hepatic and endothelial cells under flow led to the formation of functional liver organoids with enhanced metabolism compared to static conditions and increased intercellular interaction and reduced apoptosis due to the presence of HUVECs. The system was used for drug testing on a microfluidic array for high‐throughput and the integration of intestinal organoids enabled the simulation of multiorgan response to the screened drugs.^[^ ^274^ ^]^

Recently, Isshiki et al. vascularized brain organoids on a compartmentalized microfluidic device.^[^ ^276^ ^]^ Brain organoids were generated from hiPSCs, followed by coculturing with HUVECs within the microfluidic chip. The microfluidic platform had five parallel channels: one for organoid‐HUVEC coculture, which was sandwiched between two sets of microchannels where HUVECs and hLFs were suspended in cell culture media to form vasculature. Results showed that on‐chip vasculature promoted differentiation and brain organogenesis with specific in vivo features as compared to conventional monoculture. Homan et al. developed kidney organoids in perfused 3D millifluidic device (Figure 6d).^[^ ^137^ ^]^ Once harvested, organoids were introduced into the device, connected with external tubing where media was perfused through the chip via a closed loop circuit. The results showed that organoids grown under controlled high fluidic shear stress had enhanced glomerular vascularization and increase in adult gene expression as compared to organoids grown in static conditions, with development stages comparable to in vivo. Meanwhile, when organoids were grown in a prevascularized gel composed of HUVECs and hNDFs under static conditions, they were found to inhibit nephrogenesis, as compared to monoculture organoids grown under controlled flow. These findings suggest a preference for fluid flow during early stages development of kidney. The study could not prove that microvasculature formed in the kidney organoids were perfusable. Nevertheless, the feasibility to induce flow‐enhanced on‐chip organogenesis opens new strategies to form physiologically relevant in vitro models with functional vasculature. For a comprehensive review about vascularization strategies of organoids on‐chip, we refer the reader to ref. ^[^ ^277^ ^]^.

By using bioprinting‐based and microfluidic‐based fabrication methods, researchers have already successfully proved the capability to engineer complex models, as 3D printed perfusable tissue equivalents and vascularized physiologically relevant models on‐chip.^[^ ^278^, ^279^, ^280^, ^281^ ^]^ The combined use of these strategies has shown the possibility to create more reproducible and standardized constructs, laying the groundwork for the development of high throughput technologies.

## 5 Unmet Needs of Current Vascularized 3D Models

Despite the enormous progresses of the recent years, the biological complexity of vascularized 3D tissue models poses a challenge for the development of sophisticated platforms. Consequently, several limitations of the current constructs remain (**Table** **6**). Nowadays, the biological environment is recreated by 3D matrices, integration of multicellular cultures that assemble in tissue relevant structures and by providing physiologically relevant stimuli. However, cell lines are still widely used in research and ECs from umbilical vein (HUVECs) remain the top choice for endothelium modeling due to easy handling, reliability in long‐term culture, and affordable costs. Even though this common feature can be convenient when comparing results from different studies, it limits the establishment of organ‐specific models, hampering the study of tissue‐specific mechanisms at the vascular interface. Therefore, tissue specific human‐derived primary endothelial cells represent a more valid source and have been used to engineer patient‐specific platforms. However, access to human tissue and isolation protocols are often difficult and laborious operations.^[^ ^282^ ^]^ For this reason, many studies are still based on animal cell sources, which once again impede data and system scalability toward “human‐sized” models. Stem cell biology might be an alternative to address the current limitations and develop platforms for personalized medicine. Hence, vascular models using endothelial cells derived from multipotent or pluripotent stem cell sources have been already successfully engineered.^[^ ^283^, ^284^ ^]^ These cells present also the advantage of being suitable for further clinical development, such as in the case of bioprinted tissue constructs.

**Table 6:** Unmet needs of prevascularized models

Model feature	Unmet need
Model design	Difficult to replicate the capillaries size Limited examples of dense microvasculature Limited examples of thick vascularized tissues
In vitro cell culture	Extensive use of cell lines Short‐term evaluation in vitro
Environmental control	Need to integrate biochemical/ mechanical cues Need for automation Need for in situ monitoring via sensors integration

Another current limitation is the establishment of long‐term models. As presented in Table 2, 4, and 5, most of the vascularized models are used as in vitro platforms for short‐term studies (about 2 weeks) and this hampers the assessment of vascularized tissue constructs in several ways, basing on their main application. Specifically, in the case of bioprinted devices, the long‐term evaluation of their stability is fundamental for their in vivo application while, for 3D cell culture and microfluidics, the establishment of long‐term models would ensure more accurate pathology‐related and drug testing studies.^[^ ^285^, ^286^, ^287^ ^]^

The 3D geometrical complexity and the dimensions of the microcirculatory system can be more easily replicated with self‐vascularization strategies compared to prevascularization techniques due to the spontaneous assembly of ECs, with sprouts diameters often below 30 µm.^[^ ^288^, ^289^ ^]^ However, this technique is not reproducible and it takes a longer time for the vasculature to be functional and perfusable. Current bioprinting strategies have shown the capability to 3D print complex vascular geometries,^[^ ^100^, ^191^ ^]^ as well as dense tissue constructs,^[^ ^45^ ^]^ which could not be achieved otherwise. However, vessels size is still restricted by the resolution limit of many fabrication techniques and relatively few works have obtained capillary‐like diameters, mainly by laser‐based strategies, which have proved effective to create multiscale vascular networks with capillaries of less than 10 µm.^[^ ^101^, ^195^ ^]^

As discussed in Section 4.1, the incorporation of biochemical and mechanical stimuli have been successfully achieved with microfluidic‐based strategies,^[^ ^19^, ^195^ ^]^ yet engineering models that fully recapitulate the physiological cues of the microenvironment is still a challenge. In this context, 3D cell culture models such as spheroids and organoids present a solution to achieve both geometrical complexity and recapitulate the in vivo microenvironment thanks to their unique feature to self‐organize. The generation of these in vivo like constructs manifests from cell culture systems, which make it possible to amend this technology to various cell culture platforms, enabling high‐throughput screening and batch production, hence, highly translational to the industry. In terms of vascularization, spheroids/organoids present a different set of challenges. As discussed in Section 4.2, vascularized spheroids/organoids can achieve capillary‐like structures both in vitro and in vivo via coculture with ECs and transplantation in animal models. Therefore, all the technical and ethical issues associated with using ECs (cell source, availability, etc.) and animal models encompass the challenges of using vascularized spheroids/organoids for research, clinical, and industrial purposes.

The incorporation of the lymphatic system must also be considered to create more comprehensive microcirculatory models.^[^ ^290^, ^291^ ^]^ This network plays a fundamental role in tissue fluid homeostasis, immune cells trafficking, and actively participates in cardiovascular pathophysiology, cancer metastases and several diseases progression.^[^ ^292^, ^293^, ^294^ ^]^

Automation represents another key requirement in the development of reliable and high throughput platforms and, although sophisticated devices for automated manipulation, testing, and analysis on‐chip have been recently developed,^[^ ^77^, ^102^ ^]^ most of the works do not consider this feature. In parallel, the further integration of sensors for in situ monitoring of construct performances would speed up the automation, scalability, and readouts of these models, while boosting their value in both academic and industrial setups.^[^ ^201^, ^295^, ^296^, ^297^ ^]^

## 6 Industrial and Clinical Translation of Current Vascularized 3D Models

### 6.1 Scaling Up

The development of scalable vascularized models should take into account the following requirements: a reproducible, time, and cost‐effective fabrication process to obtain robust, high throughput, automated, physiologically relevant, and user‐friendly constructs or platforms.^[^ ^298^ ^]^ As aforementioned, technologies such as additive manufacturing hold potential for producing sophisticated constructs by means of reliable and rapid fabrication processes, that can be scaled‐up to mass production. However, it is important to keep in mind the need to create models that can be operated in a simple and proper way by a wide range of end‐users.

The scalability of microfluidic‐based technology is still limited by use of external bulky perfusion systems. To cope with this challenge, the multiwell format, which consists of many 3D microfluidic devices on a single plate, has been proposed successfully and produced in both academic and industrial settings.^[^ ^5^, ^299^ ^]^ This technology enables researchers to work with high throughput devices while ensuring compact designs and user‐friendly formats, conventionally used in biology and pharmaceutical fields. The multiwell format‐based and pumpless Organoplate platforms produced by the Dutch biotech company MIMETAS have been largely used for creating vascularized OOaC models and study angiogenesis without the need for external perfusion, paving the way for a tangible industrial translation of OOaC technology.^[^ ^288^, ^300^ ^]^

Organoids are considered a powerful model for drug testing and development as well as for personalized medicine. The establishment of organoids biobanks from either healthy or diseased tissues has boosted the scale‐up of this technology,^[^ ^301^, ^302^ ^]^ and protocols for large‐scale production of organoids in compliance with Good Manufacturing Practices (GMP) requirements have been recently published.^[^ ^303^, ^304^ ^]^ As discussed in Section 4.4, the use of microfluidic and bioprinting fabrication strategies could accelerate the scalability of 3D cell cultures by providing automated high throughput platforms and standardized production.^[^ ^265^ ^]^ The translation of the technology from basic research to industry and clinic poses however several challenges and questions from both the ethical and the logistic points of view. Aspects as informed consent of the donors, commercial ownership, and public versus private biobanks still need to be defined in a clear regulatory framework to enable the scale up of organoids models.^[^ ^113^, ^305^ ^]^

As for bioprinting technology, difficulties in scaling up functional tissues with adequate size to achieve vascularization limits its use for tissue repair. More importantly, questions regarding the mechanical strength and stability of bioengineered tissues, as well as their integration, innervation, immunogenicity, and maintenance of long‐term functionality after implantation, must also be considered.^[^ ^171^, ^185^ ^]^ For example, pilot studies to determine the vascularization degree of skin substitutes after in vivo implantation could contribute to the development of tissue constructs with relevant sizes to be used in the clinic but more preclinical studies are required to address such concerns.^[^ ^306^ ^]^

It is worth noting however that one of the major challenges for the scale‐up of constructs for regenerative medicine still remains the large‐scale expansion of human cells. Since billions of functional cells per patient are required for implantation,^[^ ^307^ ^]^ researchers have worked in the past years on the scalability of culture systems in line with current GMP. In this perspective, large‐scale expansion methods have moved from 2D culture systems, in which cells are expanded by multiplying the number of culture dishes, to bioreactor systems, with the advantage of introducing dynamic culturing conditions, monitoring and controlling of the culture environment, less user‐dependent variability, and higher cost and time efficiency. With the variety of bioreactors and culture methods established nowadays,^[^ ^308^ ^]^ protocols for scalable GMP production of PSCs, hiPSC‐derived cells and multipotent SCs, especially MSCs, fundamental during the angiogenesis process, have been successfully developed,^[^ ^309^, ^310^, ^311^ ^]^ although some critical aspects are still debated. For instance, media formulation still represents one of the bottlenecks and an homogenization is required, notably to prevent any unwanted differentiation during the expansion process and to cope with the high costs of the components.^[^ ^312^ ^]^ Furthermore, for the compliance with GMP standards, many other parameters, as donors selection, facilities control, storage, and distribution of the final products need to be standardized.^[^ ^313^ ^]^ The establishment of reliable and automated mass cellular production protocols is thus an essential precondition for the industrial and clinical scale‐up of tissue engineered constructs.

### 6.2 Drug Development

Drug development is a long and expensive multistep process that involves basic research and drug discovery, preclinical and clinical trials and, after the approval, postmarket monitoring. The estimated cost for the development of one new drug is of 2.5 billion dollars, of which 60% in clinical trials, and the process takes about 12 years, with less than 10% of the drug candidates succeeding in human clinical trial phases.^[^ ^10^, ^314^, ^315^ ^]^ Although the inadequacy of animals in modeling human response and related ethical issues, mammalian models are still necessary for drugs testing in preclinical phase.^[^ ^8^, ^9^, ^10^ ^]^

In this context, OOaC technology has been extensively investigated as tool to speed up drug research by better mimicking in vivo behavior and combining interactions between different tissues. Similar to spheroids/organoids, OOaC technology can lower the R&D costs and overcome the use of animal models by means of more predictive and representative preclinical systems.^[^ ^316^, ^317^, ^318^, ^319^ ^]^ Particularly, OOaC models can be used in preclinical trials for the study of PK–PD mechanisms and to test drugs already on the market for safety monitoring. The use of multiorgan‐on‐chip platforms with integrated vasculature results of particular interest for studying absorption, distribution, metabolism, excretion (ADME) pathways of new drug candidates.^[^ ^314^, ^320^ ^]^ With the European Union's full ban on testing cosmetic ingredients or products on animals in 2013, OOaC technology has emerged as well as alternative in vitro model for toxicology studies and safety assessment in cosmetics field.^[^ ^74^, ^321^ ^]^ All over the world, public and private institutions have funded OOaC‐related programs to promote and accelerate the translation of the technology from fundamental research to the industry, leading to the establishment of many OOaC start‐ups.^[^ ^315^, ^322^, ^323^ ^]^ Leading pharmaceutical and cosmetic companies are actively collaborating with some of the major start‐ups and academic centers to integrate OOaC platforms in drug testing and safety assessment in an industrial context. OOaC models have already shown higher complexity and better predictability compared to other in vitro systems. Thus, further development of these platforms to address the unmet needs could have a tremendous impact on the current drug development process.

In oncology drug research, where only ≤5% of new anticancer drug candidates is approved, tumor organoids and spheroids present a promising strategy to improve drug approval rates and serve as potential preclinical drug screening platforms.^[^ ^324^, ^325^ ^]^ For instance, colon cancer organoids were used to screen 83 drugs currently used in clinics or in clinical trials for cancer treatments. The findings demonstrated that colon cancer organoids were suitable for high throughput screening of drug candidates and could better mimic tumor microenvironment such as oxygen and nutrient gradients compared to existing models.^[^ ^324^, ^326^ ^]^ Tumor organoids have also been used successfully as preclinical models for pharmacodynamic profiling of human tumors.^[^ ^327^ ^]^ Companies like Fluofarma and InSphero offer fast‐growing 3D tumor spheroids, which can be adapted for high throughput single‐cell analysis, functional assays, drug testing, and preclinical and clinical models. Besides oncology, spheroids and organoids are also widely employed to speed up drug testing and to overcome difficulties associated with predictions of outcomes in other pathologies.^[^ ^115^, ^325^, ^328^ ^]^ InSphero develops models for diabetes and liver diseases such as nonalcoholic fatty liver disease (NAFLD) and nonalcoholic steatohepatitis (NASH) and organoids generated from ex vivo biopsy samples have been used to model genetic diseases such as cystic fibrosis (CF) for the development of precision therapies.^[^ ^329^ ^]^

The pharmaceutical industry has adopted as well bioprinted models, also due to a recent increase of the number of bioprinters on the market.^[^ ^171^ ^]^ Since 2014, liver tissue models bioprinted by Organovo are used in the pharma industry to screen liver toxicity of drugs.^[^ ^133^ ^]^ Other companies, such as Aspect Biosystems, have more recently established joint programs with pharmaceutical companies for the screening of immuno‐therapeutics to treat cancer using 3D printed models,^[^ ^330^ ^]^ as well as with multinational research organizations to develop vascularized human liver lobules by means of their microfluidic 3D bioprinting technology.^[^ ^331^ ^]^

### 6.3 Toward Clinical Application of Vascularized Models

Although recent attempts to use microfluidics and 3D cell culture constructs for tissue repair and regenerative medicine have been made,^[^ ^94^, ^332^, ^333^, ^334^, ^335^ ^]^ their application remains mainly focused on drug research and development of personalized treatments, as discussed above.^[^ ^100^, ^316^, ^326^ ^]^ Particularly, patient‐derived organoids hold great potential for transplant application since they would solve the major issues of using allogenic materials, with related immune response, and of shortage of donors.^[^ ^334^ ^]^ However, even if preclinical animal studies have shown the possible application of organoids for cell or organ transplantation, the use of models integrating vasculature remains limited.^[^ ^336^, ^337^, ^338^ ^]^ In the clinical context, bioprinting‐based vascularization strategies represent currently the most advanced technology. Intraoperative bioprinting, i.e., the direct printing of tissue on the patient in the operating theater, holds great promise together with several challenges and preclinical studies, mainly in mice, have already been successfully performed.^[^ ^339^ ^]^ Kérourédan et al. printed by LAB stem cells from apical papilla mixed with HUVEC, during surgical procedure for the treatment of murine bone calvary defect.^[^ ^214^ ^]^ The main advantage of LAB is the lack of contact between the printer and the patient tissue, when compared to extrusion methods. Nevertheless, to translate this technology to an operating room, 3D bioprinters still need to be adapted: miniaturization of the system, low printing speed, which might prolong the surgery, and the need to precisely control the light source represent important challenges.^[^ ^339^ ^]^ Besides, to assure proper vascularization of the printed tissue, 3D bioprinters should ideally print macrovessels in tandem with microvessels to enable the anastomosis with the patient circulation while ensuring instant blood supply to the construct.

Since the aim of this technology is to adapt to each patient and be performed on‐site, aspects such as standardization, customization, quality control, GMP, etc., should be defined for its application in human clinical trials. In fact, regulatory aspects for use on patients need to be defined urgently since tissues obtained by bioprinting are not yet subject to dedicated regulatory standards.^[^ ^340^, ^341^ ^]^ The elements involved in the manufacture of these tissues are i) the material, ii) the cells, iii) the software, and iv) the bioprinter. In some cases, a maturation stage is also added. Some of these elements are considered medical products (cells) and others medical devices (software), thus they would be under different regulations. The origin of the material (animal, synthetic, recombinant proteins, etc.) and cells (autologous/heterologous, embryonic, etc.), or the type of maturation (using growth factors, bioreactors, etc.) also determines the rules to follow in the different countries.^[^ ^342^ ^]^ It is therefore necessary to establish a clear framework to determine the classification of the tissues obtained by bioprinting and to define the regulatory requirements. For more information on this topic, the reader is referred to the book chapter of Li., published in 2018.^[^ ^340^ ^]^

The use of vascularized 3D models with physiological relevance can bridge the gap between in vitro research, drug development and clinical trials. Here, we have discussed how 3D cell culture models and microfluidic platforms are promising tools to improve the robustness and reliability of preclinical research data, minimize the need for animal testing and develop more efficient drug screening platforms and personalized therapies. Although their potential for transplantation and regenerative medicine has been proven, the use of complete models including vasculature is still in its infancy. On the hand, 3D bioprinting has been more widely investigated as technology for organs repair and regeneration but ethical and regulatory aspects still need to be addressed carefully to enable its safe and rapid translation.

## 7 Conclusion

The recent achievements of research in developing 3D physiological in vitro models hold promise to revolutionize the conventional regenerative medicine approaches by creating new tools for basic research, personalized medicine, drug development, and clinical application. The use of complex models integrating vasculature is a key requirement for their successful translation. Current efforts are closer than ever to engineer complex, dense and thick vascularized organ‐specific models and the continuous improvements of tissue engineering have already shown great potential in fabricating 3D physiological relevant constructs for clinical and industrial settings. Nevertheless, certain drawbacks, regarding the technical challenges, the scale‐up, and the regulatory framework still need to be addressed. On a scientific level, the combination of different and complementary tissue engineering strategies would allow researchers to overcome some of the current fabrication limits, as we have illustrated here. At the same time, the close cooperation and open dialogue of researchers, clinicians, and industry would contribute in speeding up the translational process in the near future.

## Conflict of Interest

The authors declare no conflict of interest.
